# Structural determinant for inducing RORgamma specific inverse agonism triggered by a synthetic benzoxazinone ligand

**DOI:** 10.1186/s12900-016-0059-3

**Published:** 2016-06-01

**Authors:** Douglas J. Marcotte, YuTing Liu, Kevin Little, John H. Jones, Noel A. Powell, Craig P. Wildes, Laura F. Silvian, Jayanth V. Chodaparambil

**Affiliations:** Chemical and Molecular Therapeutics, Biogen Inc, 250 Binney Street, Cambridge, MA 02142 USA; Medicinal Chemistry, Sunovion Pharmaceutical, 84 Waterford Drive, Marlborough, MA 01752 USA; Department of Brain and Cognitive Sciences, Massachusetts Institute of Technology, 77 Massachusetts Avenue, Cambridge, MA 02139 USA

**Keywords:** RORγ, Agonist, Inverse Agonist, Activation Function 2 Helix (AF2), TH17cells, IL-17, Autoimmune Disease

## Abstract

**Background:**

The nuclear hormone receptor RORγ regulates transcriptional genes involved in the production of the pro-inflammatory interleukin IL-17 which has been linked to autoimmune diseases such as rheumatoid arthritis, multiple sclerosis and inflammatory bowel disease. This transcriptional activity of RORγ is modulated through a protein-protein interaction involving the activation function 2 (AF2) helix on the ligand binding domain of RORγ and a conserved LXXLL helix motif on coactivator proteins. Our goal was to develop a RORγ specific inverse agonist that would help down regulate pro-inflammatory gene transcription by disrupting the protein protein interaction with coactivator proteins as a therapeutic agent.

**Results:**

We identified a novel series of synthetic benzoxazinone ligands having an agonist (BIO592) and inverse agonist (BIO399) mode of action in a FRET based assay. We show that the AF2 helix of RORγ is proteolytically sensitive when inverse agonist BIO399 binds. Using x-ray crystallography we show how small modifications on the benzoxazinone agonist BIO592 trigger inverse agonism of RORγ. Using an in vivo reporter assay, we show that the inverse agonist BIO399 displayed specificity for RORγ over ROR sub-family members α and β.

**Conclusion:**

The synthetic benzoxazinone ligands identified in our FRET assay have an agonist (BIO592) or inverse agonist (BIO399) effect by stabilizing or destabilizing the agonist conformation of RORγ. The proteolytic sensitivity of the AF2 helix of RORγ demonstrates that it destabilizes upon BIO399 inverse agonist binding perturbing the coactivator protein binding site. Our structural investigation of the BIO592 agonist and BIO399 inverse agonist structures identified residue Met358 on RORγ as the trigger for RORγ specific inverse agonism.

**Electronic supplementary material:**

The online version of this article (doi:10.1186/s12900-016-0059-3) contains supplementary material, which is available to authorized users.

## Background

Retinoid-related orphan receptor gamma (RORγ) is a transcription factor belonging to a sub-family of nuclear receptors that includes two closely related members RORα and RORβ. Even though a high degree of sequence similarity exists between the RORs, their functional roles in regulation for physiological processes involved in development and immunity are distinct [1]. During development, RORγ regulates the transcriptional genes involved in the functioning of multiple pro-inflammatory lymphocyte lineages including T helper cells (TH17cells) which are necessary for IL-17 production [2]. IL-17 is a pro-inflammatory interleukin linked to autoimmune diseases such as rheumatoid arthritis, multiple sclerosis and inflammatory bowel disease; making its transcriptional regulation through RORγ an attractive therapeutic target [3–5].

RORγ consists of an N-terminal DNA binding domain (DBD) connected to a C-terminal ligand binding domain (LBD) via a flexible hinge region. The DBD is composed of two zinc fingers that allow it to interact with specifically encoded regions on the DNA called the nuclear receptor response elements. The LBD consists of a coactivator protein binding pocket and a hydrophobic ligand binding site (LBS) which are responsible for regulating transcription. The coactivator binding pocket of RORγ recognizes a conserved helix motif LXXLL (where X can be any amino acid) on transcriptional coactivator complexes and recruits it to activate transcription [6]. Like other nuclear hormone receptors, RORγ’s helix12 which makes up the C-termini of the LBD is an essential part of the coactivator binding pocket and is commonly referred to as the activation function helix 2 (AF2) [7]. In RORγ, the conformation of the AF2 helix required to form the coactivator binding pocket is mediated by a salt bridge between His479 and Tyr502 in addition to π- π interactions between Tyr502 and Phe506 [8]. The conformation of the AF2 helix can be modulated through targeted ligands which bind the LBS and increase the binding of the coactivator protein (agonists) or disrupt binding (inverse agonists) thereby enhancing or inhibiting transcription [1, 6]. Since RORγ has been demonstrated to play an important role in pro-inflammatory gene expression patterns implicated in several major autoimmune diseases, our aim was to develop RORγ inverse agonists that would help down regulate pro-inflammatory gene transcription [1–5, 9–12].

**Fig. 1:**
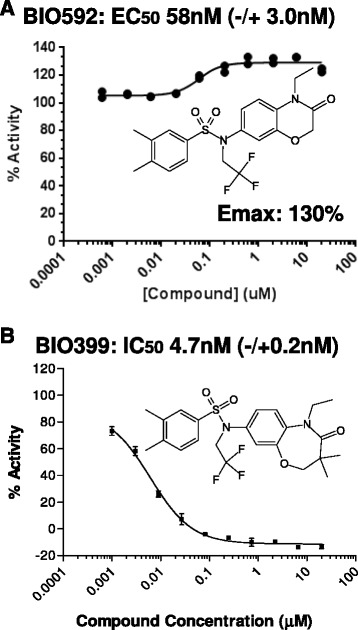
FRET results for agonist BIO592 (**a**) and Inverse Agonist BIO399 (**b**)

Here we present the identification of two synthetic benzoxazinone RORγ ligands, a weak agonist BIO592 (Fig. 1a) and an inverse agonist BIO399 (Fig. 1b) which were identified using a Fluorescence Resonance Energy transfer (FRET) based assay that monitored coactivator peptide recruitment. Using partial proteolysis in combination with mass spectrometry analysis we demonstrate that the AF2 helix of RORγ destabilizes upon BIO399 (inverse agonist) binding. Finally, comparing binding modes of our benzoxazinone RORγ crystal structures to other ROR structures, we hypothesize a new mode of action for achieving inverse agonism and selectivity.

## Methods

### Cloning, protein expression and purification of RORγ518

GST-RORγ518 was constructed by sub-cloning residues 259 to 518 of a human RORγ cDNA into a pGEX-6P vector with a cleavable N-terminal GST fusion tag. BL21 (DE3) *Escherichia coli* cells were transformed with the plasmid encoding the GST-PreScission-hRORgamma 259–518 protein (GST-RORγ518) and were grown at 37 °C in LB media supplemented with ampicillin to an OD of 1. The temperature was reduced to 18 °C and protein expression was induced by adding 1 mM IPTG and was shaking for an additional 16 h. The cells were harvested and resuspended in lysis buffer (25 mM TRIS pH 8.0, 250 mM NaCl, 10 % Glycerol, 5 mM DTT and Roche EDTA-free protease inhibitor cocktail) and were lysed using a microfluidizer. The lysate was clarified by centrifugation at 20,000 × g for 1 h at 4 °C and GST-RORγ518 was captured by batch binding to Glutathione Sepharose resin overnight at 4 °C. The resin was washed with buffer A (25 mM TRIS pH 8.0, 250 mM NaCl, 10 % glycerol, 5 mM DTT) and loaded onto a XK column and washed until no non-specific unbound protein was detected. GST- RORγ518 was eluted from the column using buffer A supplemented with 10 mM Glutathione pH 8.0 and analyzed by SDS-PAGE. The eluate was then treated with PreScission Protease (10units/mg of protein) and further purified on a Superdex 75 column equilibrated in buffer B (25 mM TRIS pH 8.0, 250 mM NaCl, 5 % glycerol and 2 mM DTT). RORγ518 eluted as a monomer and was approximately 95 % pure as observed by SDS-PAGE.

Additional constructs including c-terminal truncations, surface entropy reduction and cysteine scrubbed mutations were also expressed and purified in the same manner as RORγ518 if an expression level of >1 mg/L was achieved.

### RORγ FRET based assay and GAL4 reporter assay

FRET-based (Fluorescence Resonance Energy Transfer) assay and the GAL4 Reporter assay were performed as described previously [13]. BIO592 and BIO399 were synthesized (Additional file 1) and belonged to a proprietary library where they were identified as RORγ activity modulators using the FRET-based assay.

### Partial proteolysis of RORγ518

RORγ518 at 8 mg/ml or in complex with 1 mM BIO399 or 1 mM BIO592 and 0.5 mM coactivator peptide EBI96 EFPYLLSLLGEVSPQ (New England Peptide) were treated with Actinase E (Hampton Research) added at a ratio of 1.25ugs of protease/1 mg of RORγ518 for 6 h at 4 °C [14]. The reactions were quenched using 1X Protease inhibitor cocktail (Roche) + 1 mM EDTA and subjected to mass spectrometry analysis.

### Mass spectrometry of partially proteolyzed RORγ518

Proteolyzed RORγ518 samples were reduced with 50 mM dithiothreitol in 50 mM Tris pH 8.0, 150 mM NaCl containing 4 M urea and 5 mM EDTA. The sample was then analyzed on a LC-MS system comprised of a UPLC (ACQUITY, Waters Corp.), a TUV dual-wavelength UV detector (Waters Corp.), and a ZQ mass spectrometer (Waters Corp.). A Vydac C4 cartridge was used for desalting. Molecular masses for the Actinase E treated RORγ518 samples were obtained by deconvoluting the raw mass spectra using MaxLynx 4.1 software (Waters Corp.).

### Crystallization of RORγ518 with agonist BIO592 and inverse agonist BIO399

RORγ518 was concentrated to 8 mg/ml and EBI96 was added to a final concentration of 0.5 mM and agonist BIO592 to 1 mM and incubated on ice for 1 h. The coactivator peptide EBI96 which was identified by phage display was chosen for crystallization because of its strong interaction with RORγ in a mammalian two-hybrid analysis system that assessed the transactivation of RORγ [14]. Diffraction quality crystals were grown through vapor diffusion in a buffer containing 0.1 M HEPES pH 8.0, 25 % PEG3350 and 0.2 M NaCl at 18 °C. Crystals were cryoprotected in the mother liquor containing 20 % glycerol as cryoprotectant prior to being frozen in liquid nitrogen for data collection.

Actinase E proteolyzed RORγ518 BIO399 concentrated to 8 mg/ml was crystallized using vapor diffusion in a buffer containing 0.1 M BisTRIS pH 5.5, 0.2 M ammonium acetate and 15 % PEG3350 at 18 °C. Crystals were cryoprotected for data collection by transferring them to a mother liquor containing 15 % PEG400 prior to being frozen in liquid nitrogen.

### Data collection and structure determination for RORγ518 BIO592 and BIO399 complexes

X-ray diffraction data for all the crystals were measured at beam line ID31 at the Argonne Photon Source. The data were processed with Mosflm [15] in case of the RORγ518-BIO592-EBI96 ternary complex and with HKL2000 [16] in the case of the Actinase E treated aeRORγ518/BIO399 complex. For both datasets, PDB ID: 3LOL [17] was used as the search model, and the molecular replacement solutions were determined using MOLREP [18]. The refinement was carried out using Refmac5 [19] and model building was carried out in Coot [20]. The data processing and refinement statistics are provided in Additional file 2.

RORγ518-BIO592-EBI96 ternary complex:

The data for the ternary complex were measured to 2.63 Å. It crystallized in a P21 space group with four molecules of the ternary complex in the asymmetric unit. The final model was refined to a R_cryst_ of 19.9 % and R_free_ of 25.5 %.

aeRORγ518/BIO399 complex:

Diffraction data for the aeRORγ518-BIO399 complex were measured to 2.35 Å. It crystallized in C2 space group with two molecules in the asymmetric unit. The final model was refined to a R_cryst_ of 21.1 % and R_free_ of 26.3 %.

## Results and discussion

### Identification of BIO592 and BIO399 as ligands that modulate RORγ coactivator peptide recruitment

Using a FRET based assay we discovered agonist BIO592 (Fig. 1a) which increased the coactivator peptide TRAP220 recruitment to RORγ (EC_50_ 0f 58nM and E_max_ of 130 %) and a potent inverse agonist BIO399 (Fig. 1b) which inhibited coactivator recruitment (IC_50_: 4.7nM). Interestingly, the structural difference between the agonist BIO592 and inverse agonist BIO399 was minor; with the 2,3-dihydrobenzo[1,4]oxazepin-4-one ring system of BIO399 being 3 atoms larger than the benzo[1,4]oxazine-3-one ring system of BIO592. In order to understand how small changes in the core ring system leads to inverse agonism, we wanted to structurally determine the binding mode of both BIO592 and BIO399 in the LBS of RORγ using x-ray crystallography.

### Structure of the RORγ518-BIO592-EBI96 ternary complex is in a transcriptionally active conformation

**Fig. 2:**
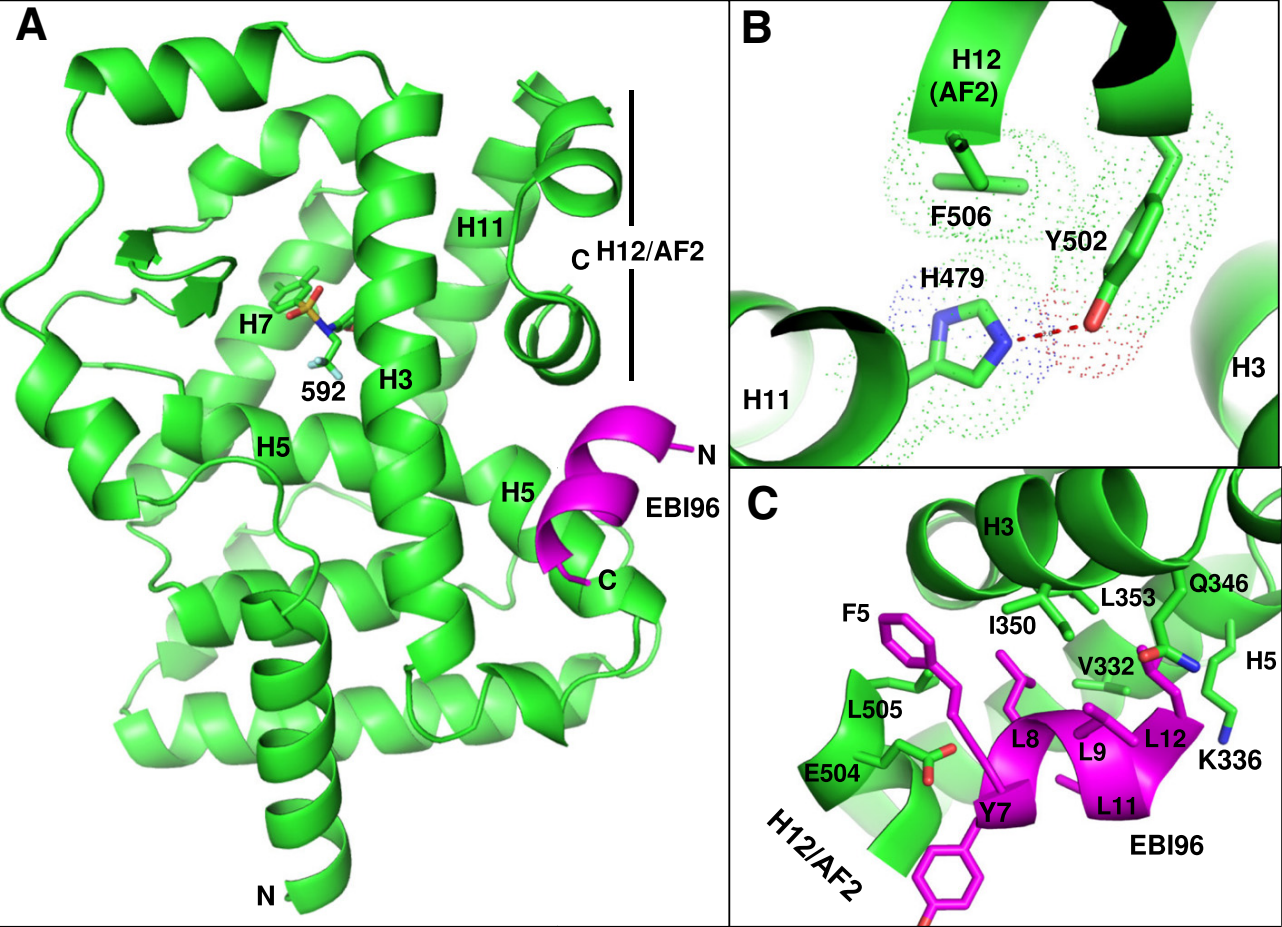
**a** The ternary structure of RORγ518 BIO592 and EBI96. **b** RORγ AF2 helix in the agonist conformation. **c** EBI96 coactivator peptide bound in the coactivator pocket of RORγ

RORγ518 bound to agonist BIO592 was crystallized with a truncated form of the coactivator peptide EBI96 to a resolution of 2.6 Å (Fig. 2a). The structure of the ternary complex had features similar to other ROR agonist coactivator structures in a transcriptionally active canonical three layer helix fold with the AF2 helix in the agonist conformation [21]. The agonist conformation is stabilized by a hydrogen bond between His479 and Tyr502, in addition to π-π interactions between His479, Tyr502 and Phe506 (Fig. 2b). The hydrogen bond between His479 and Tyr502 has been reported to be critical for RORγ agonist activity. Disrupting this interaction through mutagenesis reduced transcriptional activity of RORγ [14]. This reduced transcriptional activity has been attributed to the inability of the AF2 helix to complete the formation of the coactivator binding pocket necessary for coactivator proteins to bind.

Electron density for the coactivator peptide EBI96 was observed for residues EFPYLLSLLG which formed a α-helix stabilized through hydrophobic interactions with the coactivator binding pocket on RORγ (Fig. 2c). This interaction is further stabilized through a conserved charged clamp wherein the backbone amide of Tyr7 and carbonyl of Leu11 of EBI96 form hydrogen bonds with Glu504 (helix12) and Lys336 (helix3) of RORγ. Formation of this charged clamp is essential for RORγ’s function for playing a role in transcriptional activation and this has been corroborated through mutagenic studies in this region [14, 17].

### BIO592 binds in a collapsed conformation stabilizing the agonist conformation of RORγ

**Fig. 3:**
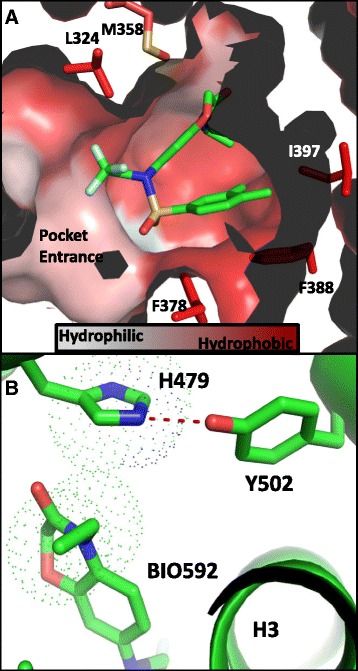
**a** Collapsed binding mode of agonist BIO592 in the hydrophobic LBS of RORγ. **b** Benzoxazinone ring system of agonist BIO592 packing against His479 of RORγ stabilizing agonist conformation of the AF2 helix

BIO592 bound in a collapsed conformational state in the LBS of RORγ with the xylene ring positioned at the bottom of the pocket making hydrophobic interactions with Val376, Phe378, Phe388 and Phe401, with the ethyl-benzoxazinone ring making several hydrophobic interactions with Trp317, Leu324, Met358, Leu391, Ile 400 and His479 (Fig. 3a, Additional file 3). The sulfonyl group faces the entrance of the pocket, while the CF_3_ makes a hydrophobic contact with Ala327. Hydrophobic interaction between the ethyl group of the benzoxazinone and His479 reinforce the His479 sidechain position for making the hydrogen bond with Tyr502 thereby stabilizing the agonist conformation (Fig. 3b).

### RORγ AF2 helix is sensitive to proteolysis in the presence of Inverse Agonist BIO399

Next, we attempted co-crystallization with the inverse agonist BIO399. However, extensive crystallization efforts with BIO399 and RORγ518 or other AF2 intact constructs did not produce crystals. We hypothesized that the RORγ518 coactivator peptide interaction in the FRET assay was disrupted upon BIO399 binding and that a conformational rearrangement of the AF2 helix could have occurred, hindering crystallization.

**Fig. 4:**
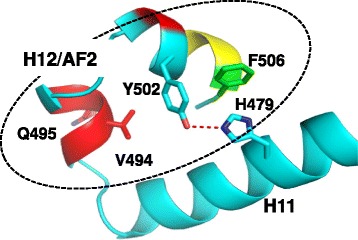
Specific proteolytic positions on RORγ518 when treated with Actinase E alone (Green) or in the presence of BIO399 (Red) and shared proteolytic sites (Yellow)

The unfolding of the AF2 helix has been observed for other nuclear hormone receptors when bound to an inverse agonist or antagonist [22–24]. We used partial proteolysis in combination with mass spectrometry to determine if BIO399 was causing the AF2 helix to unfold [25]. Results of the Actinase E proteolysis experiments on RORγ518, the ternary complex of RORγ518 with agonist BIO592 and coactivator EBI96, or in the presence of inverse agonist BIO399 supported our hypothesis. Analysis of the fragmentation pattern showed minimal proteolytic removal of the AF2 helix by Actinase E on RORγ518 alone (ending at 504 to 506) and the ternary complex remained primarily intact (ending at 515/518) (Additional file 4). However, in the presence of inverse agonist BIO399, the proteolytic pattern showed significantly less protection, albeit the products were more heterogeneous (majority ending at 494/495), indicating the destabilization of the AF2 helix compared to either the APO or ternary agonist complex (Fig. 4, Additional file 5).

Several rounds of cocrystallization attempts with RORγ518 or other RORγ AF2 helix containing constructs complexed with BIO399 had not produced crystals. We attributed the inability to form crystals to the unfolding of the AF2 helix induced by BIO399. We reasoned that if we could remove the unfolded AF2 helix using proteolysis we could produce a binary complex more amenable to crystallization.

### AF2 truncated RORγ BIO399 complex is more amenable to crystallization

**Fig. 5:**
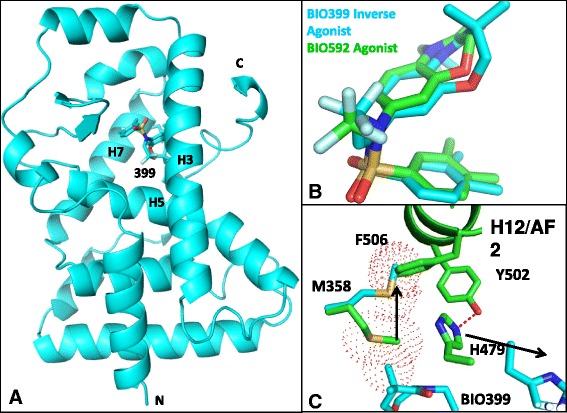
**a** The binary structure of AF2-truncated RORγ and BIO399. **b** The superposition of inverse agonist BIO399 (Cyan) and agonist BIO592 (Green). **c** Movement of Met358 and His479 in the BIO399 (Cyan) and BIO592 (Green) structures

The Actinase E treated RORγ518 BIO399 ternary complex (aeRORγ493/4) co-crystallized readily in several PEG based conditions. The structure of aeRORγ493/4 BIO399 complex was solved to 2.3 Å and adopted a similar core fold to the BIO592 agonist crystal structure (Fig. 5a, Additional file 3). The aeRORγ493/4 BIO399 structure diverged at the c-terminal end of Helix 11 from the RORγ518 BIO592 EBI96 structure, where helix 11 unwinds into a random coil after residue L475.

### Inverse agonist BIO399 uses Met358 as a trigger for inverse agonism

BIO399 binds to the ligand binding site of RORγ adopting a collapsed conformation as seen with BIO592 where the two compounds superimpose with an RMSD of 0.72 Å (Fig. 5b). The majority of the side chains within 4 Å of BIO399 and BIO592 adopt similar rotomer conformations with the exceptions of Met358 and His479 (Fig. 5c). The difference density map showed clear positive density for Met358 in an alternate rotomer conformation compared to the one observed in the molecular replacement model or the other agonist containing models (Additional file 6). We tried to refine Met358 in the same conformation as the molecular replacement model or the other agonist containing models, but the results clearly indicated that this was not possible, thus confirming the new rotamer conformation for the Met358 sidechain in the inverse agonist bound structure. The change in rotomer conformation of Met358 between the agonist and inverse agonist structures is attributed to the gem-dimethyl group on the larger 7 membered benzoxazinone ring system of BIO399. The comparison of the two structures shows that the agonist conformation observed in the BIO592 structure would be perturbed by BIO399 pushing Met358 into Phe506 of the AF2 helix indicating that Met358 is a trigger for inducing inverse agonism in RORγ (Fig. 5c).

### BIO399 and Inverse agonist T0901317 bind in a collapsed conformation distinct from other RORγ Inverse Agonists Cocrystal structures

**Fig. 6:**
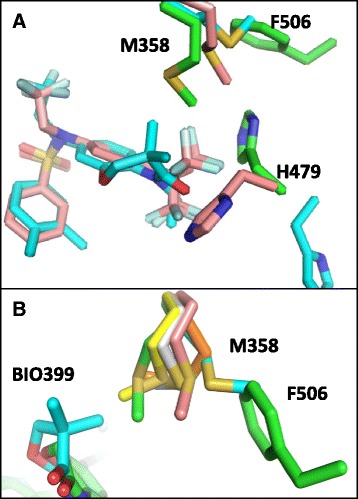
**a** Overlay of RORγ structures bound to BIO596 (Green), BIO399 (Cyan) and T0901317 (Pink). **b** Overlay of M358 in RORγ structure BIO596 (Green), BIO399 (Cyan), Digoxin (Yellow), Compound 2 (Grey), Compound 48 (Salmon) and Compound 4j (Orange)

The co-crystal structure of RORγ with T0901317 (PDB code: 4NB6), an inverse agonist of RORγ (IC_50_ of 54nM in an SRC1 displacement FRET assay and an IC_50_ of 59nM in our FRET assay (Additional file 7)) shows that it adopts a collapsed conformation similar to the structure of BIO399 described here [26]. The two compounds superimpose with an RMSD of 0.81 Å (Fig. 6a). The CF3 group on the hexafluoropropanol group of T0901317 was reported to fit the electron density in two conformations one of which pushes Met358 into the vicinity of Phe506 in the RORγ BIO592 agonist structure. We hypothesize that since the Met358 sidechain conformation in the T0901317 RORγ structure is not in the BIO399 conformation, this difference could account for the 10-fold reduction in the inverse agonism for T0901317 compared to BIO399 in the FRET assay.

Co-crystal structures of RORγ have been generated with several potent inverse agonists adopting a linear conformation distinct from the collapsed conformations seen for BIO399 and T090131718 [27–31]. The inverse agonist activity for these compounds has been attributed to orientating Trp317 to clash with Tyr502 or a direct inverse agonist hydrogen bonding event with His479, both of which would perturb the agonist conformation of RORγ. BIO399 neither orients the sidechain of Trp317 toward Tyr502 nor forms a hydrogen bond with His479 suggesting its mode of action is distinct from linear inverse agonists (Additional file 8). In the linear inverse agonist crystal structures the side chain of Met358 resides in a similar position as the rotomer observed in RORγ agonist structures with BIO592 described here or as observed in the hydroxycholesterol derivatives and therefore would not trigger inverse agonism with these ligands (Fig. 6b) [17].

### BIO399 shows selectivity for RORγ over RORα and RORβ in a GAL4 Cellular Reporter Assay

**Fig. 7:**
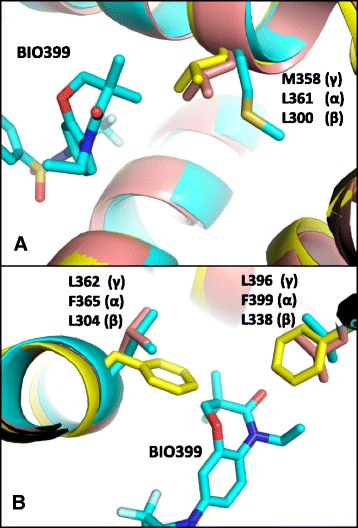
**a** Overlay of RORα (yellow), β (pink) and γ (cyan) showing side chain differences at Met358 inverse agonism trigger position and (**b**) around the benzoxazinone ring system of BIO399

In order to assess the in vivo selectivity profile of BIO399 a cellular reporter assay was implemented where the ligand binding domains of ROR α, β and γ were fused to the DNA binding domain of the transcriptional factor GAL4. The ROR-GAL4 fusion proteins were expressed in cells with the luciferase reporter gene under the control of a GAL4 promoter [13]. BIO399 inhibited the luciferase activity when added to the cells expressing the RORγ-GAL4 fusion with an in vivo IC_50_ of 42.5nM while showing >235 and 28 fold selectivity over cells expressing GAL4 fused to the LBD of ROR α or β, respectively (Table 1). The LBS of RORs share a high degree of similarity. However, the inverse agonism trigger of BIO399, residue Met358, is a leucine in both RORα and β. This selectivity profile for BIO399 is attributed to the shorter leucine side chain in RORα and β which would not reach the phenylalanine on the AF2 helix further underscoring the role of Met358 as a trigger for RORγ specific inverse agonism (Fig. 7a). Furthermore, RORα contains two phenylalanine residues in its LBS whereas RORβ and γ have a leucine in the same position (Fig. 6b). We hypothesize that the two phenylalanine residues in the LBS of RORα occlude the dihydrobenzoxazepinone ring system of BIO399 from binding it and responsible for the increase in selectivity for RORα over β.

**Table 1:** GAL4 cell assay selectivity profile for BIO399 toward RORα and RORβ in GAL4

ROR	γ	α	β
IC50 (uM)	0.043 (+/− 0.01uM; N = 6)	>10 (N = 2)	>1.2 (N = 2)
Selectivity (X)	-	>235	>28.2

## Conclusions

We have identified a novel series of synthetic benzoxazinone ligands which modulate the transcriptional activity of RORγ in a FRET based assay. Using partial proteolysis we show a conformational change which destabilizes the AF2 helix of RORγ when the inverse agonist BIO399 binds. The two RORγ co-crystal structures reported here show how a small change to the core ring system can modulate the mode of action from agonist (BIO592) to inverse agonism (BIO399). Finally, we are reporting a newly identified trigger for achieving RORγ specific inverse agonism in an in vivo setting through Met358 which perturbs the agonist conformation of the AF2 helix and prevents coactivator protein binding.

## Abbreviations

AF2, activation function 2; BisTRIS, 2-[Bis(2-hydroxyethyl)amino]-a-(hydroxymethyl)propane-1,3-diol; DND, DNA binding domain; DTT, 1,4-Dithiothreitol; EDTA, 2-({2-[Bis(carboxymethyl)amino]ethyl}(carboxymethyl)amino)acetic acid; FRET, fluorescence resonance energy transfer; GST, Glutathione-S-Transferase; HEPES, 2-[4(2-hydroxyethyl)-1-piperazineethanesulfonic acid; IC_50_, half maximal inhibitory concentration; IL-17, Interleukin-17; IPTG, isopropyl β-D-1-thiogalactopyranoside; LBD, Ligand Binding Domain; LBS, ligand binding site; LC-MS, liquid chromatography/mass spectrometry; PDB, Protein Data Bank; ROR, retinoid orphan receptor; SRC-1, steroid receptor coactivator-1; TH17 Cells, T helper cells; TRIS, 2-amino-2-hydroxymethyl-propane-1,3,diol.

## Additional files

Additional file 1: Synthesis route and spectra data for compounds BIO399 and BIO592. (DOCX 54 kb) Additional file 2: Crystallography table of refinement statistics for the RORγ structures with BIO592 and BIO399. (PDF 37 kb) Additional file 3: 2Fo-Fc electron density for BIO592 and BIO399 in the ligand binding site of RORγ. (PDF 549 kb) Additional file 4: Mass spectrometry results for RORγ and Actinase E treated RORγ. (PDF 4625 kb) Additional file 5: Positions of Actinase E proteolysis sites for APO, Ternary BIO592 EBI96 complex and the BIO399 binary complex determined by mass spectrometry. (PDF 30 kb) Additional file 6: Difference density at 2σ for the position of Met358 in the RORγ molecular replacement model PDB: 3L0L) and refined in the RORγ BIO399 structure. (PDF 343 kb) Additional file 7: RORγ FET assay results for T0901317 and DMSO control replicates. (PDF 34 kb) Additional file 8: Superposition of Trp317, Met358 and His479 side chains in the RORγ BIO592 agonist structure and linear inverse agonist structures. (PDF 525 kb)
