# Supplementary material for: Structural determinant for inducing RORgamma specific inverse agonism triggered by a synthetic benzoxazinone ligand
Source: BMC Struct Biol. 2016 Jun 1;16:7. doi: 10.1186/s12900-016-0059-3 (PMC4888278; doi:10.1186/s12900-016-0059-3)
Supplement: Additional file 1: — Synthesis route and spectra data for compounds BIO399 and BIO592. (DOCX 54 kb) [file 12900_2016_59_MOESM1_ESM.docx]

**Synthesis of BIO592 and BIO399**

**N-(4-Ethyl-3-oxo-3,4-dihydro-2H-benzo[b][1,4]oxazin-7-yl)-3,4-dimethylbenzenesulfonamide:**

7-Amino-4-ethyl-2H-benzo[b][1,4]oxazin-3(4H)-one (163.2 mg, 0.85 mmol) was dissolved in a mixture of CH_2_Cl_2_ (9.0 ml) and pyridine (1.10 ml) and the solution cooled to 0^°^C. 3,4-Dimethyl-benzenesulfonyl chloride (205 mg, 1.0 mmol) was added in a dropwise fashion. After the addition was complete, the ice bath was removed, and the reaction was stirred at room temperature for 2 hr. The mixture was diluted with water and extracted with EtOAc. The organic phase were separated, and solvent was removed under reduced pressure to afford N-(4-ethyl-3-oxo-3,4-dihydro-2H-benzo[b][1,4]oxazin-7-yl)-3,4-dimethylbenzenesulfonamide in quantitative yield, which was used without further purification. ESI-MS 361.1 [M+H].

**N-(4-ethyl-3-oxo-3,4-dihydro-2H-benzo[b][1,4]oxazin-7-yl)-3,4-dimethyl-N-(2,2,2-trifluoroethyl)benzenesulfonamide (BIO592):**

N-(4-Ethyl-3-oxo-3,4-dihydro-2H-benzo[b][1,4]oxazin-7-yl)-3,4-dimethylbenzenesulfonamide (307 mg, 0.85 mmol) and Trifluoro-methanesulfonic acid 2,2,2-trifluoro-ethyl ester (340 mg, 0.85 mmol) were dissolved in a solution of K_2_CO_3_ (353 mg, 2.6 mmol) in CH_3_CN (12 ml, 220 mmol). The reaction was heated to 55^°^C and stirred overnight. The reaction mixture was cooled to room temperature and concentrated to dryness under reduced pressure. Purification by reverse phase HPLC gave N-(4-ethyl-3-oxo-3,4-dihydro-2H-benzo[b][1,4]oxazin-7-yl)-3,4-dimethyl-N-(2,2,2-trifluoroethyl)benzenesulfonamide **BIO592** (281 mg, 77%) as a white powder. ESI-MS: 443.0 [M+H]. ^1^H NMR (400 MHz, DMSO-d_6_) δ 7.49 (d, *J*=1.25 Hz, 1H), 7.28-7.37 (m, 2H), 7.18 (d, *J*=8.78 Hz, 1H), 6.80 (dd, *J*=2.51, 8.78 Hz, 1H), 6.71 (d, *J*=2.26 Hz, 1H), 4.64 (s, 2H), 4.51 (q, *J*=8.95 Hz, 2H), 3.90 (q, *J*=7.03 Hz, 2H), 2.31 (s, 3H), 2.28 (s, 3H), 1.06-1.18 (m, 3H).

**N-(9-Ethyl-7,7-dimethyl-8-oxo-6,7,8,9-tetrahydro-5-oxa-9-aza-benzocyclohepten-
3-yl)-3,4-dimethyl-benzenesulfonamide:**

3-Amino-9-ethyl-7,7-dimethyl-6,7-dihydro-9H-5-oxa-9-aza-benzocyclohepten-8-one (170.4 mg, 0.73 mmol) was dissolved in a mixture of CH_2_Cl_2_ (7.5 ml) and pyridine (0.94 ml, 11.6 mmol) and the solution cooled to 0^0^C. After dropwise addition of 3,4-dimethyl-benzenesulfonyl chloride (179 mg, 0.83 mmol), the ice bath removed, and the reaction mixture was stirred for 2 hr. The mixture was poured into water and extracted with EtOAc. The organics were separated and concentrated under reduced pressure to yield N-(9-Ethyl-7,7-dimethyl-8-oxo-6,7,8,9-tetrahydro-5-oxa-9-aza-benzocyclohepten-3-yl)-3,4-dimethyl-benzenesulfonamide in quantitative yield, which was used without further purification. ESI-MS: 403.1 [M+H].

**N-(9-Ethyl-7,7-dimethyl-8-oxo-6,7,8,9-tetrahydro-5-oxa-9-aza-benzocyclohepten-
3-yl)-3,4-dimethyl-N-(2,2,2-trifluoro-ethyl)-benzenesulfonamide (BIO399):**

N-(9-Ethyl-7,7-dimethyl-8-oxo-6,7,8,9-tetrahydro-5-oxa-9-aza-benzocyclohepten-
3-yl)-3,4-dimethyl-benzenesulfonamide (292 mg, 0.73 mmol) and Trifluoro-methanesulfonic acid 2,2,2-trifluoro-ethyl ester (0.115 ml, 0.80 mmol) were dissolved in a suspension of K_2_CO_3_ (303 mg, 2.2 mmol) in CH_3_CN (10 ml, 190 mmol). The suspension was heated to 55^°^C and stirred overnight. The reaction was cooled to room temperature and concentrated to dryness under reduced pressure. Purification by reverse phase HPLC afforded N-(9-Ethyl-7,7-dimethyl-8-oxo-6,7,8,9-tetrahydro-5-oxa-9-aza-benzocyclohepten-3-yl)-3,4-dimethyl-N-(2,2,2-trifluoro-ethyl)-benzenesulfonamide **BIO399** (246 mg, 71%) as a white powder. ESI-MS: 458.2

[M+H]. ^1^H NMR (400 MHz, DMSO-d6): δ 7.43 (s, 1H), 7.32 - 7.39 (m, 2H), 7.26 - 7.32 (m, 1H), 7.06 (dd, *J* = 2.13, 8.66 Hz, 1H), 6.78 (d, *J* = 2.26 Hz, 1H), 4.56 (q, *J* = 8.78 Hz, 2H), 4.06 (s, 2H), 3.81 (q, *J* = 6.94 Hz, 2H), 2.31 (s, 3H), 2.26 (s, 3H), 1.04 (t, *J* = 6.90 Hz, 3H), 0.90 (s, 6H).
