# Supplementary material for: Structural determinant for inducing RORgamma specific inverse agonism triggered by a synthetic benzoxazinone ligand
Source: BMC Struct Biol. 2016 Jun 1;16:7. doi: 10.1186/s12900-016-0059-3 (PMC4888278; doi:10.1186/s12900-016-0059-3)
Supplement: Additional file 2: — Crystallography table of refinement statistics for the RORγ structures with BIO592 and BIO399. (PDF 37 kb) [file 12900_2016_59_MOESM2_ESM.pdf]

**Additional file 2.** Collection and refinement statistics for RORγ structures.

| Data Collection | EBI96/BIO592 | BIO399 |
|---|---|---|
| Space Group | $P2_1$ | C2 |
| Cell Dimensions | | |
| a (Å) | 85 | 126 |
| b (Å) | 68 | 56 |
| c (Å) | 96 | 78 |
| Wavelength (Å) | 0.98 | 0.98 |
| Resolution (Å) | 2.63 | 2.35 |
| $R_{sym}^{a}$ | 0.109 (0.449) | 0.10 (0.437) |
| I/σ | 9.9 (2.7) | 16.2 (2.8) |
| Multiplicity | 3.7 (3.7) | 3.3 (3.3) |
| Total No. reflections/ No. unique reflections | 114,149/31,077 | 36,027/18,850 |
| Mean I/σ | 9.9/2.7 | 16.2/2.8 |
| Completeness (%) | 99.6 (99.7) | 99.5 (99.9) |
| Rwork/Rfree | 19.9/25.5 | 21.1/26.3 |
| $CC_{1/2}$ | 0.95 (0.58) | 0.97 (0.66) |
| No. Molecules per asymmetric unit | 4 | 2 |
| R.m.s.d. bond distance (Å) | 0.016 | 0.021 |
| R.m.s.d bond angle (deg) | 1.65 | 2.1 |
| Total no. of non-H atoms in ASU | 8,528 | 3,574 |
| No. of solvent molecules | 171 | 12 |
| Avg. protein B-value (Å$^2$) | 32.5 | 49.7 |
| Avg. solvent B-value (Å$^2$) | 34.2 | 54.5 |
| Ramachandran Plot | | |
| Preferred | 977 | 417 |
| Generous | 23 | 13 |
| Disallowed | 1 | 1 |

***The value in parentheses is for the highest resolution bin (approximate interval, 0.1 A)**

**[a]Rsym = ƒ°| ƒ§hkl.< ƒ§hkl >|/ ƒ° ƒ§hkl**

**[b]Rwork = ƒ°ƒ§hkl| |Fo . |Fc| | / ƒ°ƒ§hkl|Fo| for all data except 5% which is used for the Rfree calculation**
