# Supplementary figures and images for: Structural determinant for inducing RORgamma specific inverse agonism triggered by a synthetic benzoxazinone ligand
Source: BMC Struct Biol. 2016 Jun 1;16:7. doi: 10.1186/s12900-016-0059-3 (PMC4888278; doi:10.1186/s12900-016-0059-3)

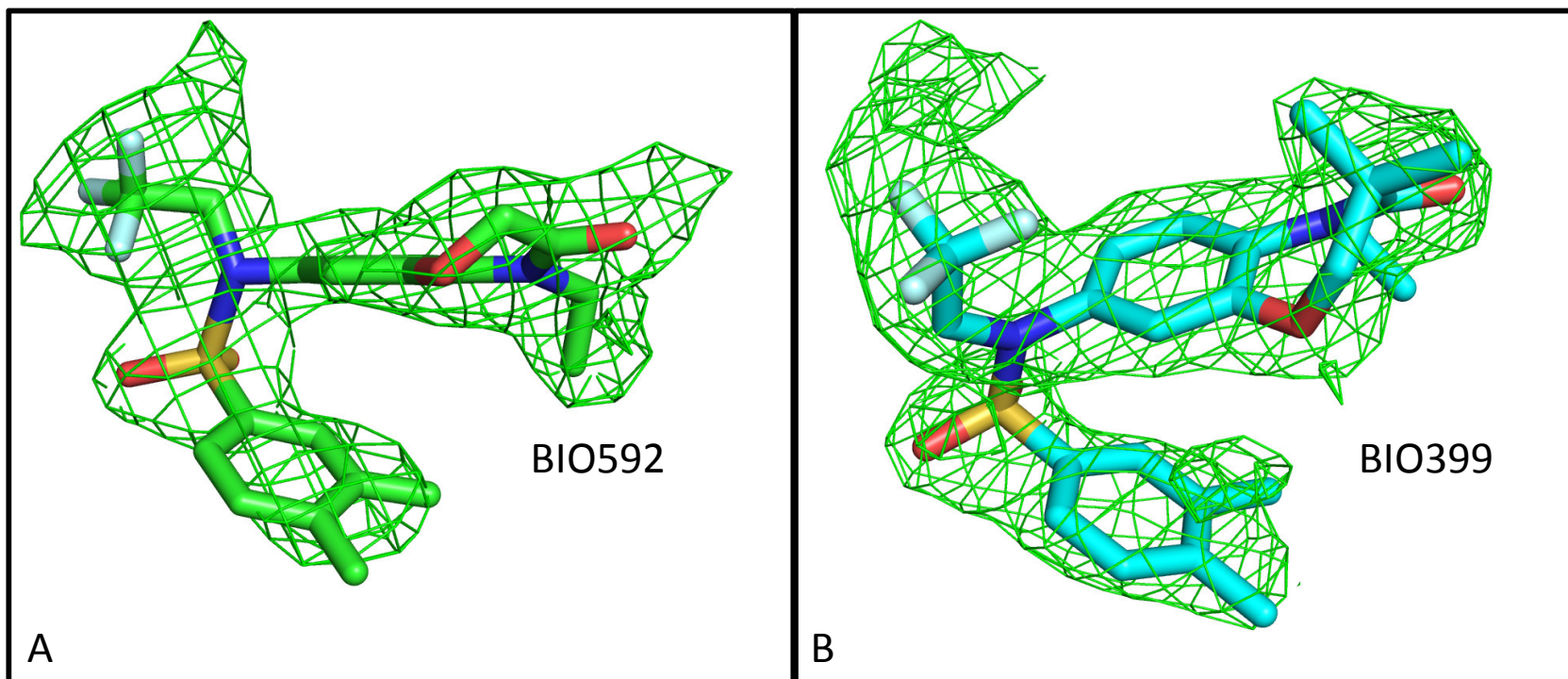

**Additional file 5:** 2fo-fc electron density contoured at  $2\sigma$  for A) BIO592 and B) BIO399.

Supplement: Additional file 3: — 2Fo-Fc electron density for BIO592 and BIO399 in the ligand binding site of RORγ. (PDF 549 kb) [file 12900_2016_59_MOESM3_ESM.pdf]

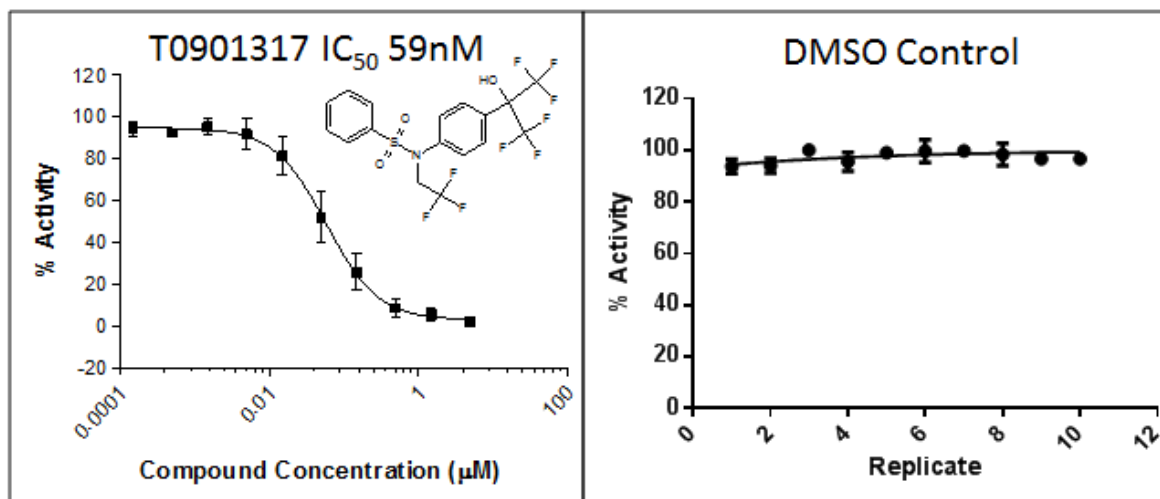

**Additional File 7:** ROR $\gamma$  FET assay results for T0901317 and DMSO control replicates.

Supplement: Additional file 7: — RORγ FET assay results for T0901317 and DMSO control replicates. (PDF 34 kb) [file 12900_2016_59_MOESM7_ESM.pdf]
