# Supplementary material for: Structural determinant for inducing RORgamma specific inverse agonism triggered by a synthetic benzoxazinone ligand
Source: BMC Struct Biol. 2016 Jun 1;16:7. doi: 10.1186/s12900-016-0059-3 (PMC4888278; doi:10.1186/s12900-016-0059-3)
Supplement: Additional file 4: — Mass spectrometry results for RORγ and Actinase E treated RORγ. (PDF 4625 kb) [file 12900_2016_59_MOESM4_ESM.pdf]

AF2 reduction paper figures

Additional file 2. Statistics of data and structures

Data Collection	EBI96/BIO592	BIO399
Space Group	$P2_1$	C2
Cell Dimensions		
a (Å)	85	126
b (Å)	68	56
c (Å)	96	78
Wavelength (Å)	0.98	0.98
Resolution (Å)	2.63	2.35
$R_{sym}{}^{a}$	0.109 (0.449)	0.10 (0.437)
I/σ	9.9 (2.7)	16.2 (2.8)
Multiplicity	3.7 (3.7)	3.3 (3.3)
Total No. reflections/ No. unique reflections	114,149/31,077	36,027/18,850
Mean I/σ	9.9/2.7	16.2/2.8
Completeness (%)	99.6 (99.7)	99.5 (99.9)
Rwork/Rfree	19.9/25.5	21.1/26.3
$CC_{1/2}$	0.95 (0.58)	0.97 (0.66)
No. Molecules per asymmetric unit	4	2
R.m.s.d. bond distance (Å)	0.016	0.021
R.m.s.d bond angle (deg)	1.65	2.1
Total no. of non-H atoms in ASU	8,528	3,574
No. of solvent molecules	171	12
Avg. protein B-value (Å^2)	32.5	49.7
Avg. solvent B-value (Å^2)	34.2	54.5
Ramachandran Plot		
Preferred	977	417
Generous	23	13
Disallowed	1	1

***The value in parentheses is for the highest resolution bin (approximate interval, 0.1 A)**

[a]Rsym = ƒ°| ƒ§hkl.< ƒ§hkl >|/ ƒ° ƒ§hkl

[b]Rwork = ƒ°ƒ§hkl| |Fo . |Fc| | / ƒ°ƒ§hkl|Fo| for all data except 5% which is used for the Rfree calculation

*The value in parentheses is for the highest resolution bin (approximate interval, 0.1 A)
Rsym = ƒ°| ƒ§hkl.< ƒ§hkl >|/ ƒ° ƒ§hkl
aRwork = ƒ°ƒ§hkl| |Fo . |Fc| | / ƒ°ƒ§hkl|Fo| for all data except 5% which is used for the Rfree calculation

sup1

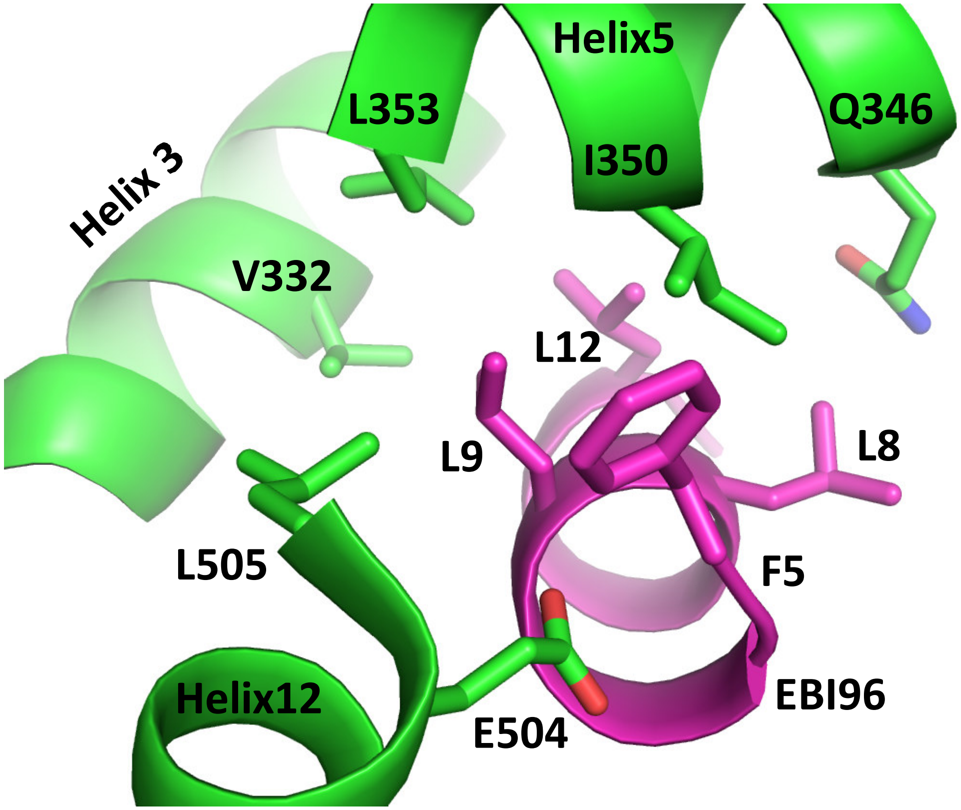
Helix5
L353
Q346
I350
Helix 3
V332
L12
L8
L9
L505
F5
EBI96
Helix12
E504

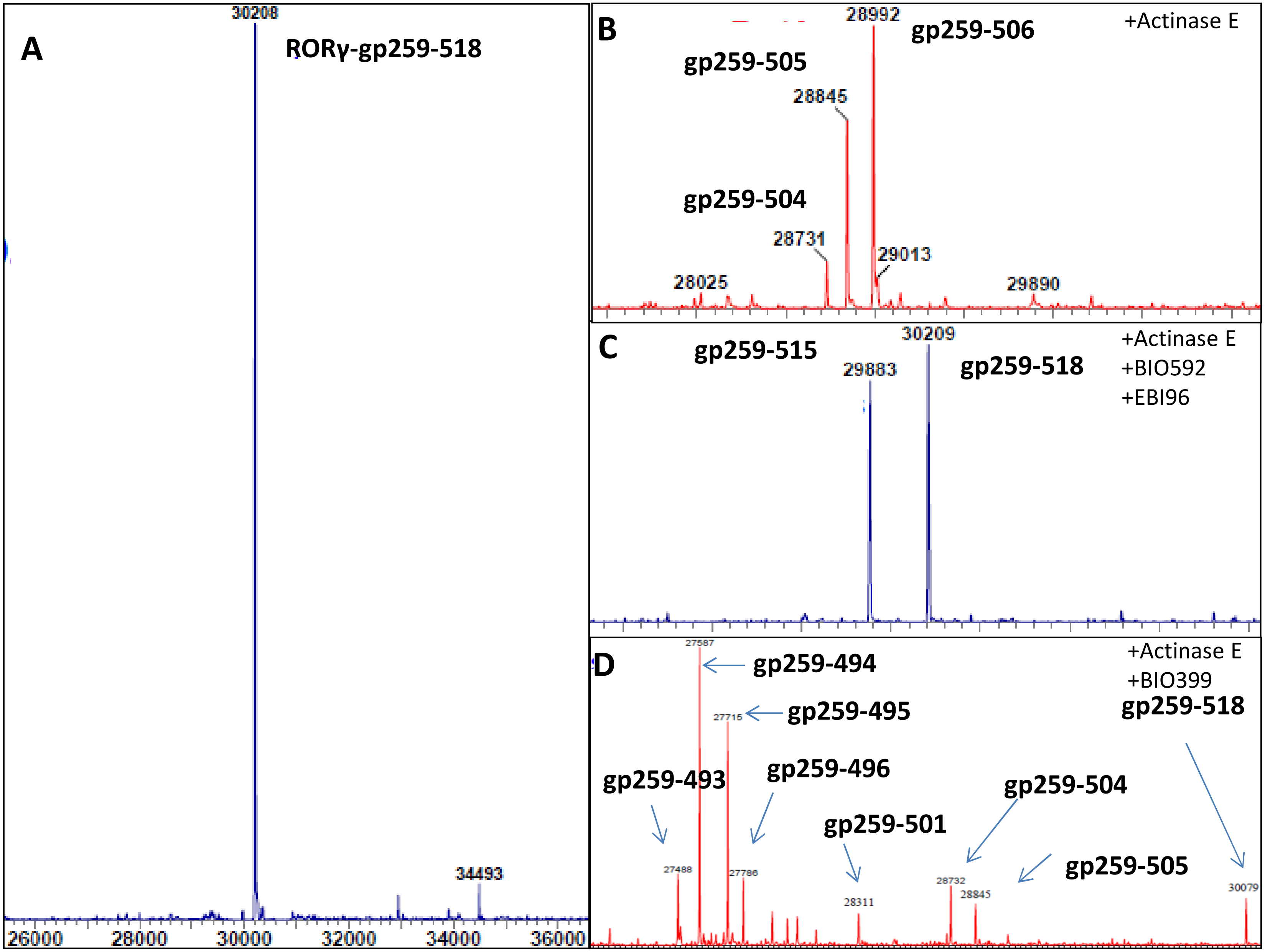

Additional File 3: Mass spectrometry results for A) RORγ518, B) RORγ518 treated with Actinase E, C) RORγ518 BIO592 and EBI96 complex treated with Actinase E and D) RORγ518 BIO399 complex treated with ActinaseE.

RORγ518	Actinase E	C-terminal Cleavage site(s)
APO	-	518
APO	+	504, 505, 506
BIO592 + EBI96	+	518, 515
BIO399	+	493, 494, 495, 496, 501, 504, 505, 518

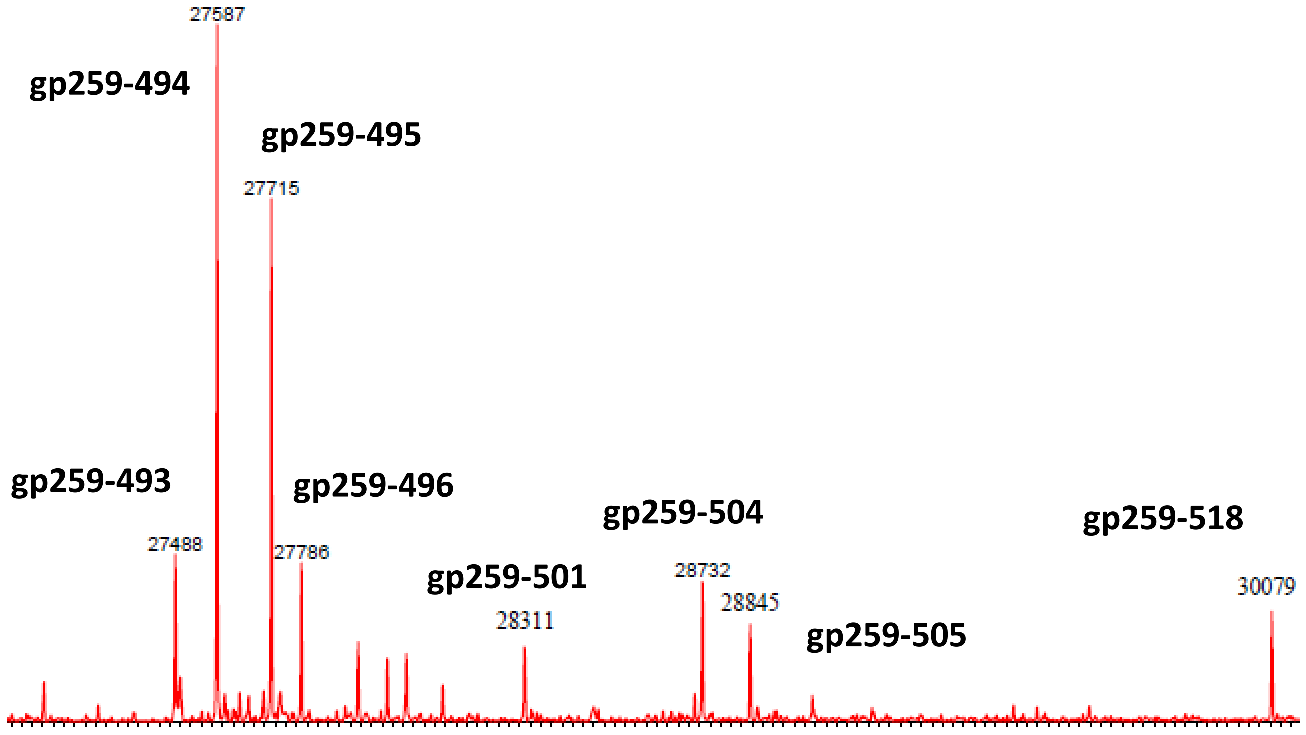

27587
gp259-494
gp259-495
27715
gp259-493
27488
gp259-496
27786
gp259-501
28311
gp259-504
28732
28845
gp259-505
gp259-518
30079

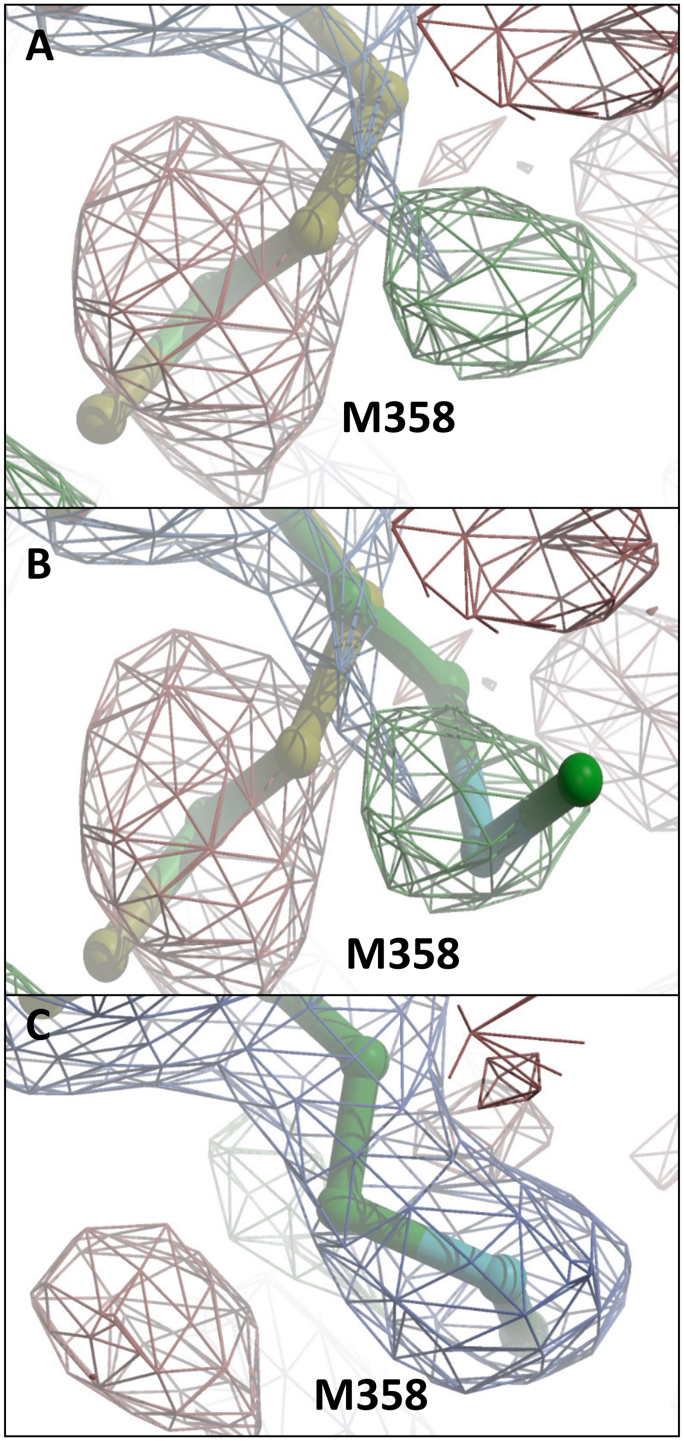

Sup3

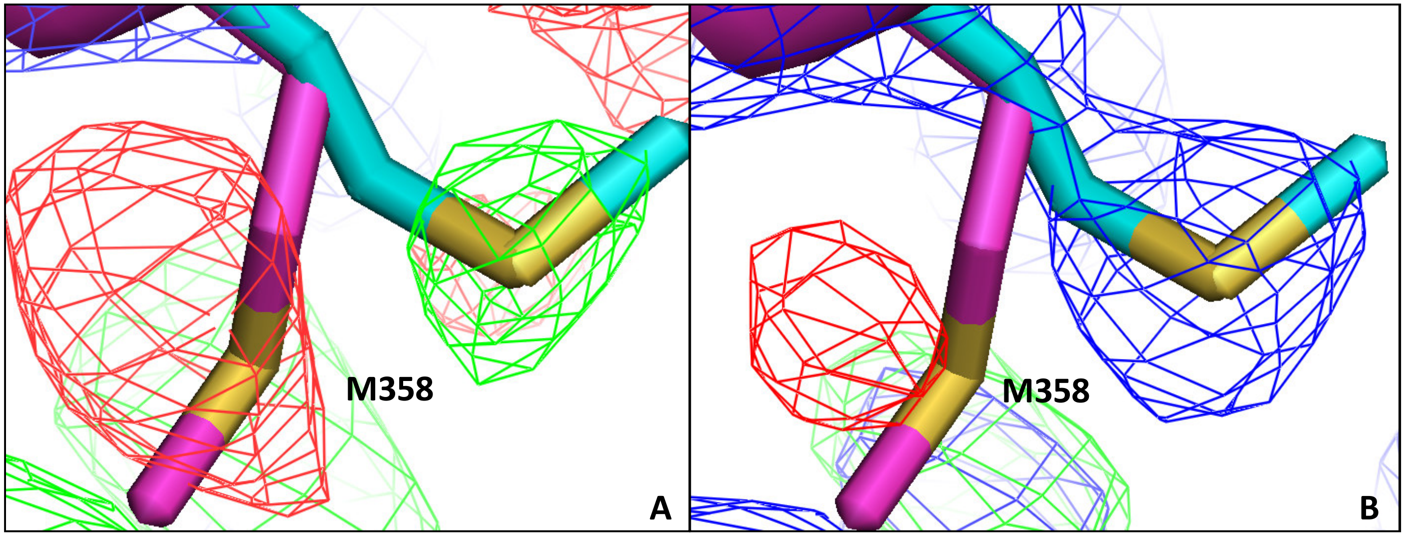
M358
A
M358
B

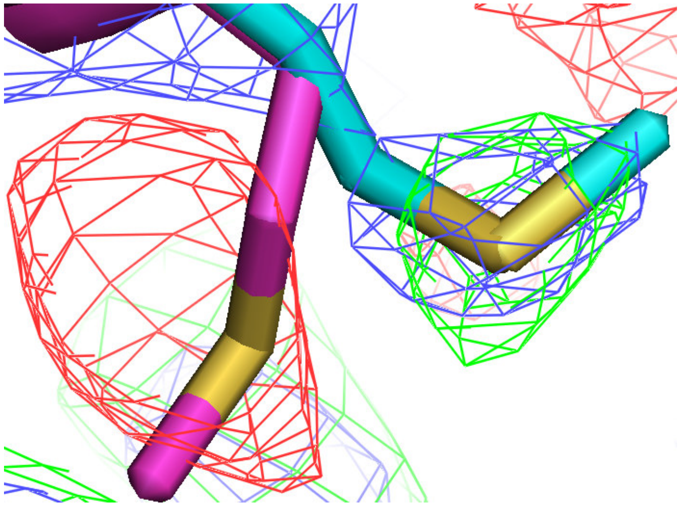

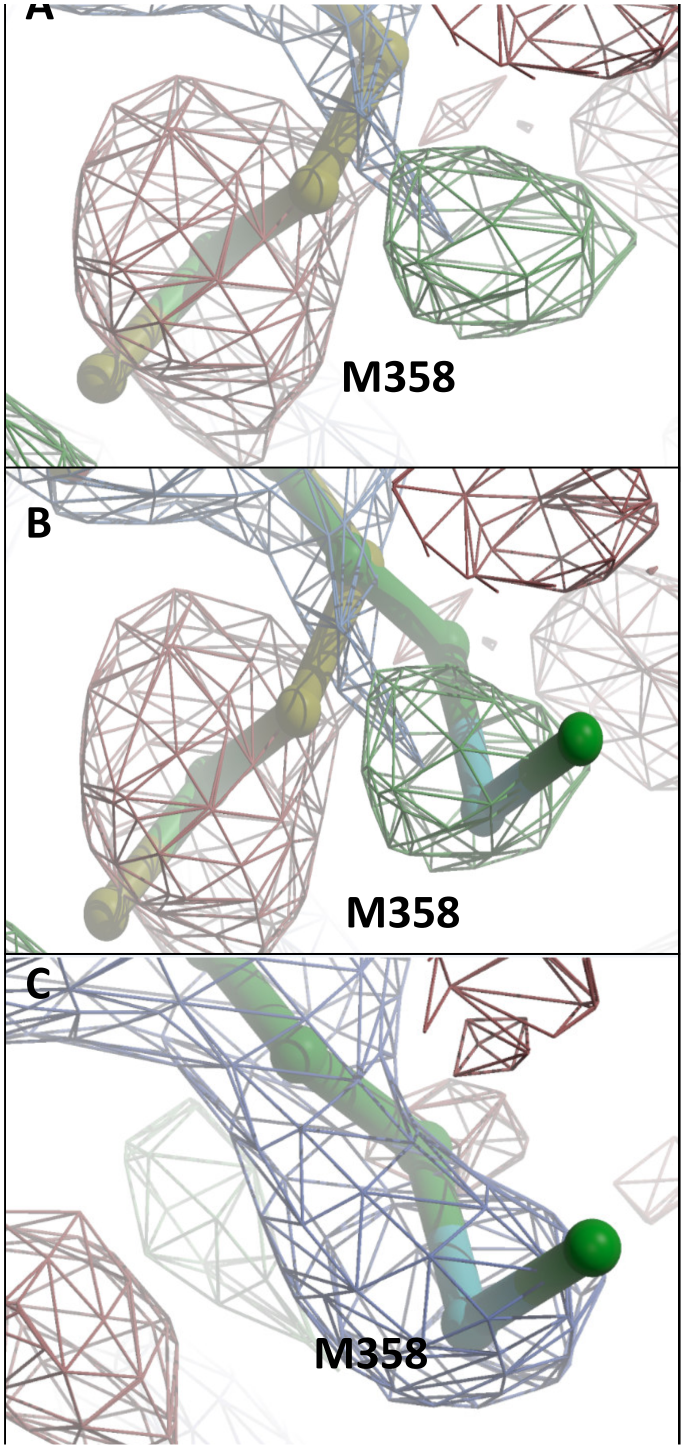
A
M358
B
M358
C
M358

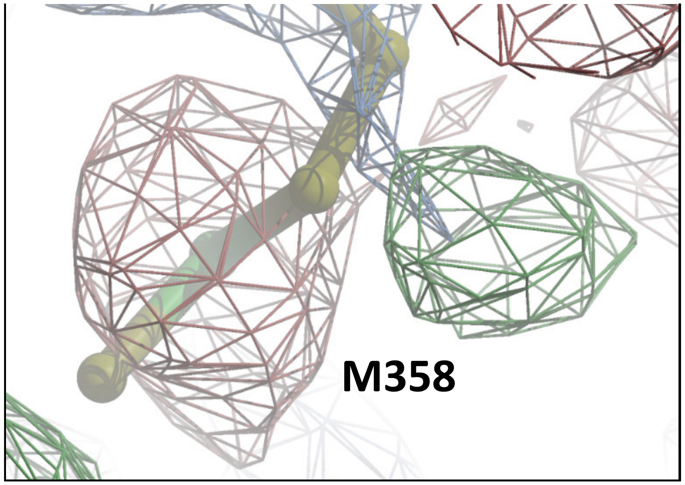
M358

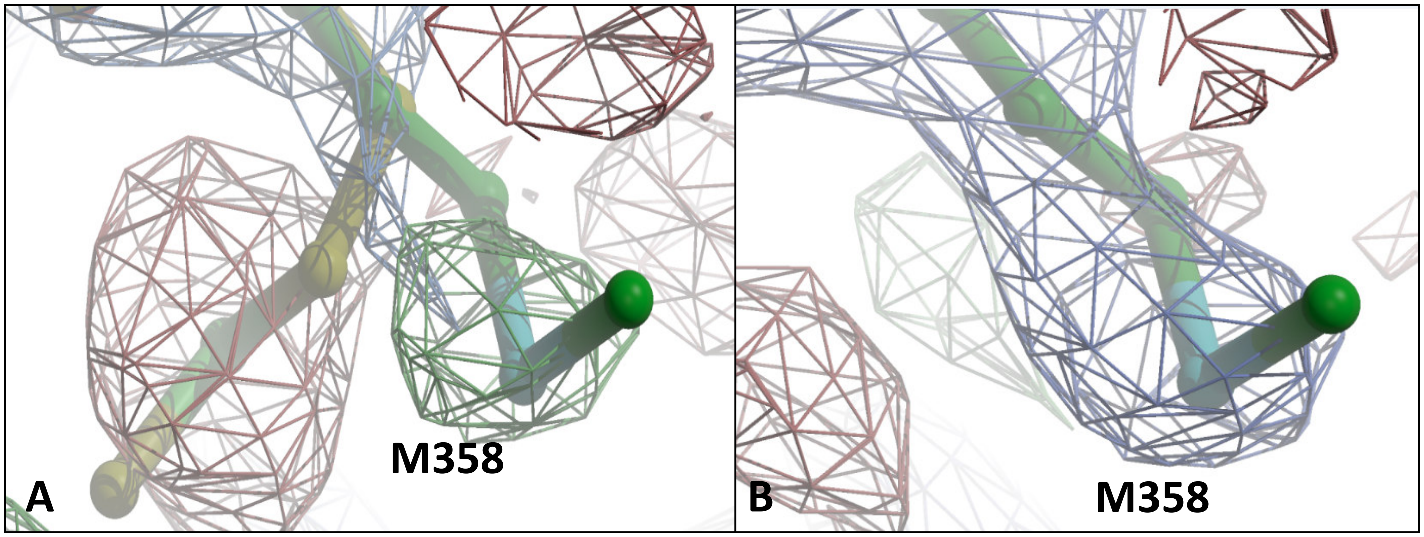
M358
A
M358
B

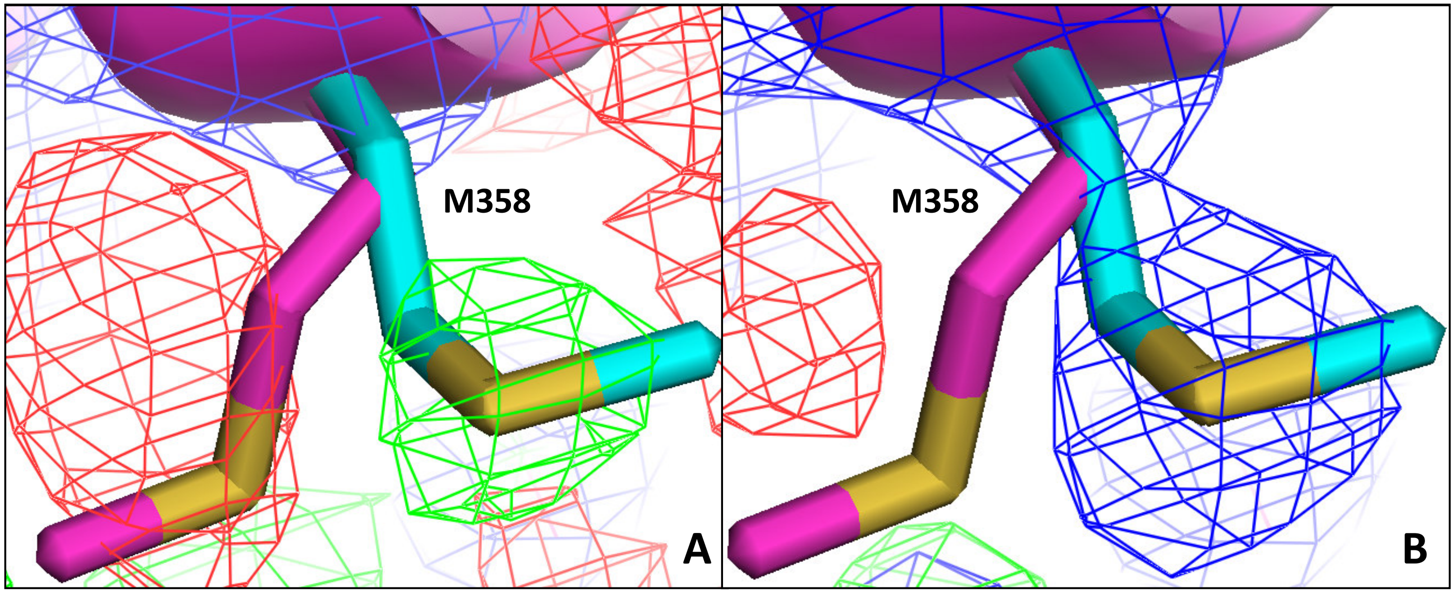

Supplementary Figure 3: Position of Met358 in the RORγ BIO399 binary structure. A) Initial difference density at 2σ for Met358 from molecular replacement model 3L0L after first round of refinement (magenta) and alternate rotomer conformation (cyan). B) Electron density for Met358 rotomer after second round of refinement.

RORγ518	Actinase E	C-terminal Cleavage site(s)
APO	-	518
APO	+	504, 505, 506
BIO592 + EBI96	+	518, 515
BIO399	+	494, 495, 504, 505, 506

Table 2: Positions of Actinase E proteolysis sites for APO, Ternary BIO592 EBI96 complex and BIO399 determined by mass spectrometry.

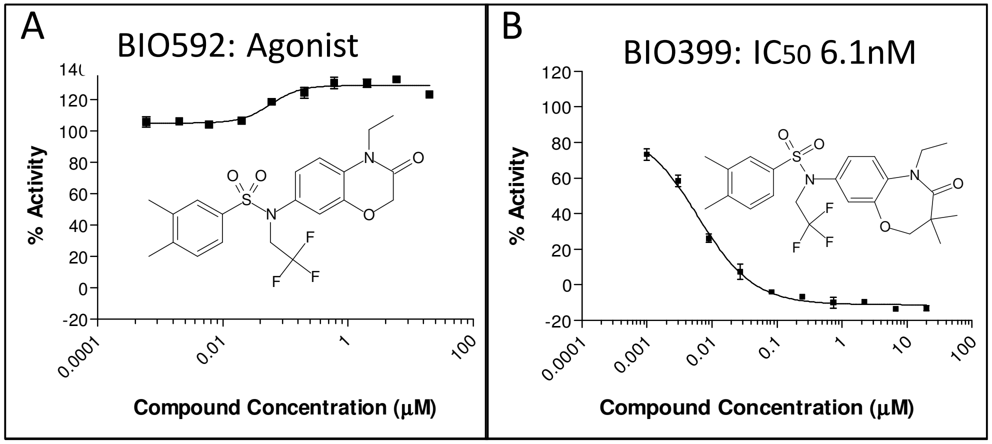

Figure1: FRET results for Agonist BIO592 (A) Inverse Agonist BIO399 (B).

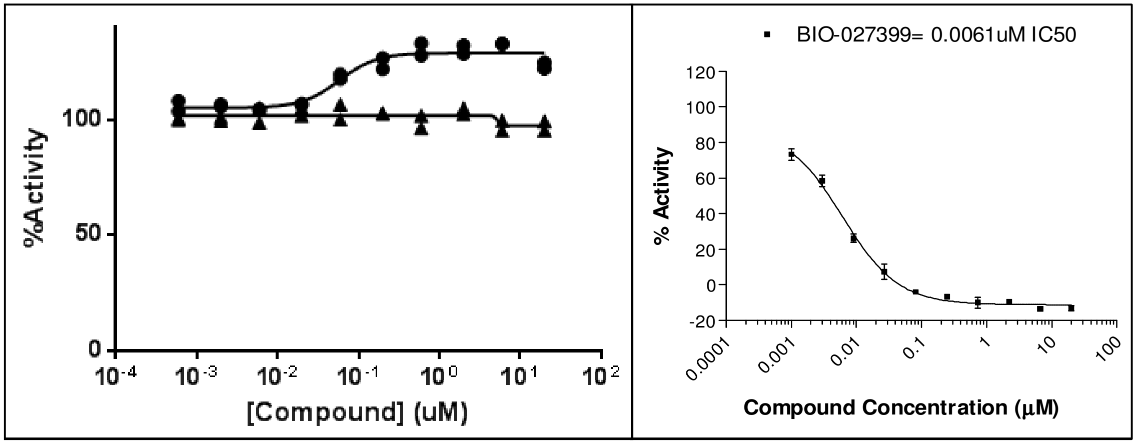

%Activity
100
50
0
10^-4
10^-3
10^-2
10^-1
10^0
10^1
10^2
[Compound] (uM)
BIO-027399= 0.0061uM IC50
% Activity
120
100
80
60
40
20
0
-20
0.0001
0.001
0.01
0.1
1
10
100
Compound Concentration (μM)

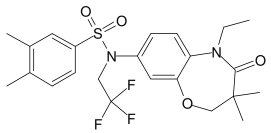

O
O
S
N
N
O
F
F
F
O

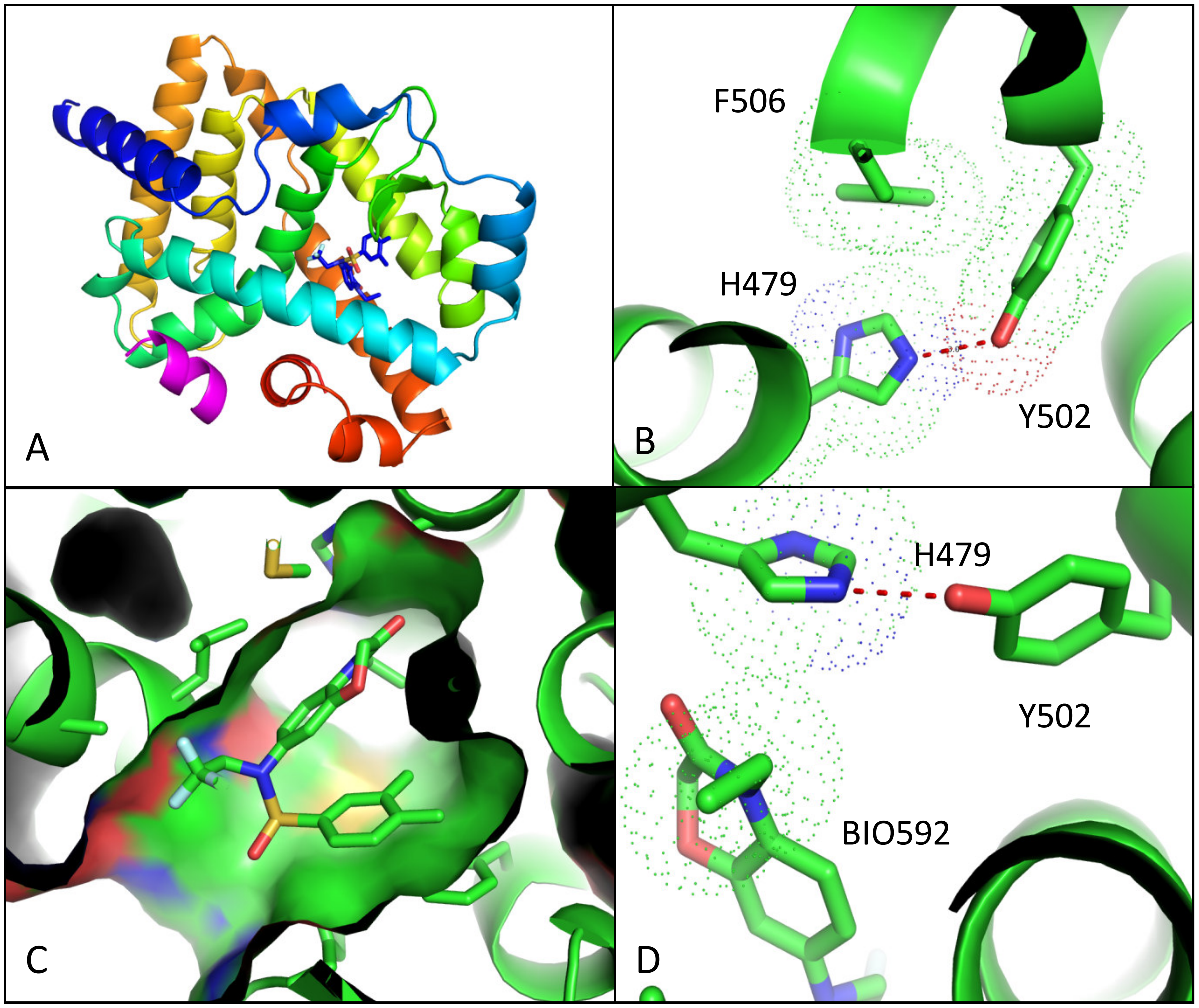

Figure 2: A) The ternary structure of RORγ518 BIO592 and EBI96. B) Agonist Conformation of AF2 helix in the ternary structure. C) Binding mode of BIO592 in the LBS of RORγ. D) BIO592 benzoxazinone ethyl engages His479.

A

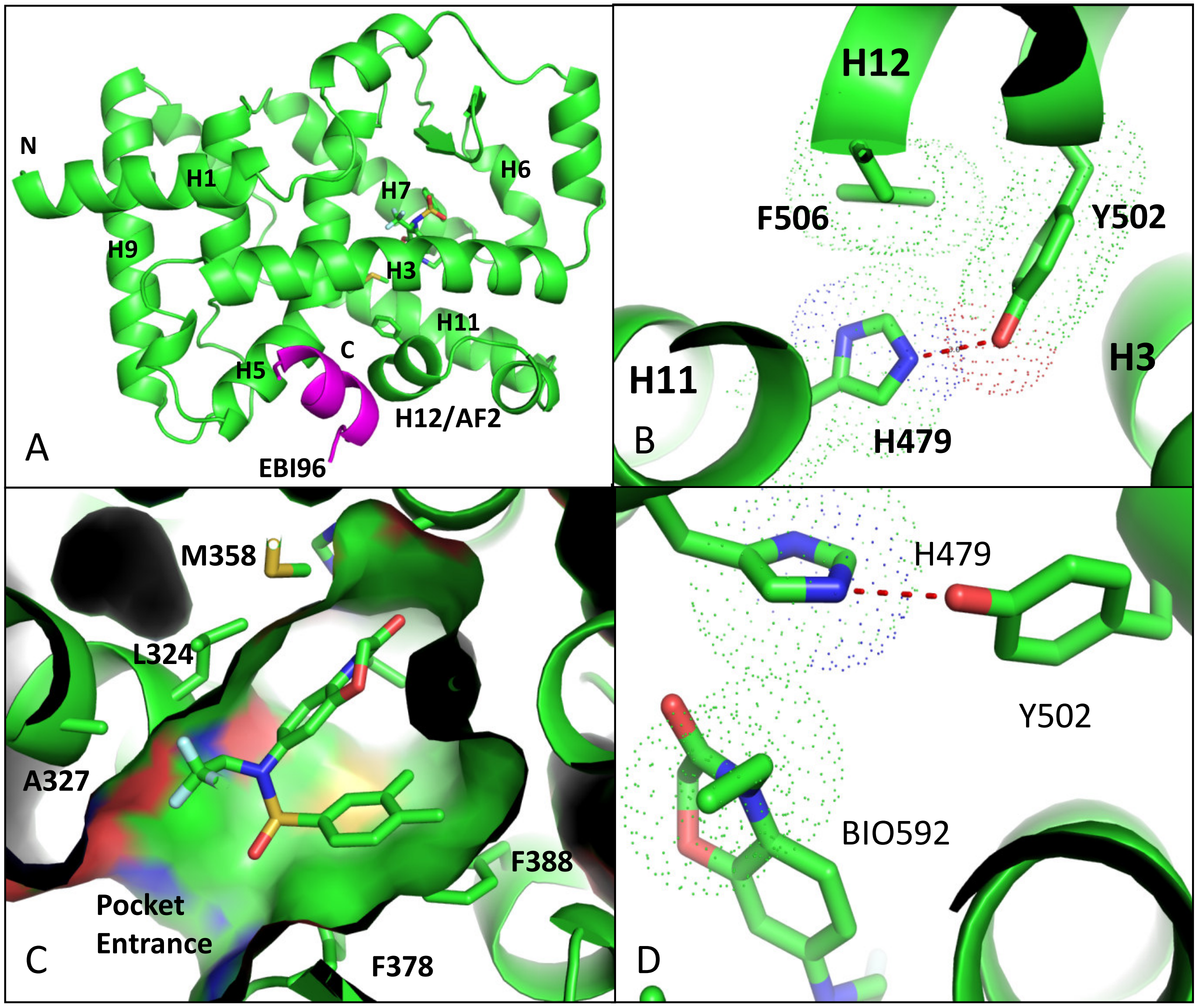
N
H1
H9
H7
H6
H3
H11
C
H5
H12/AF2
EBI96
A
H12
F506
Y502
H11
H3
H479
B
M358
L324
A327
F388
Pocket
Entrance
F378
C
H479
Y502
BIO592
D

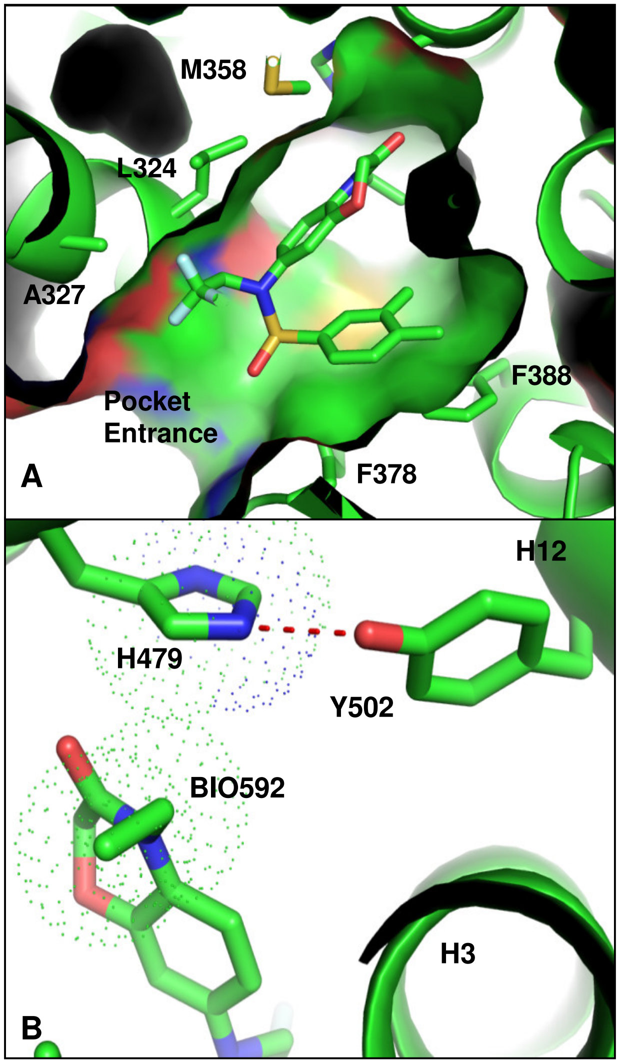
M358
L324
A327
Pocket
Entrance
F388
F378
A
H12
H479
Y502
BIO592
H3
B

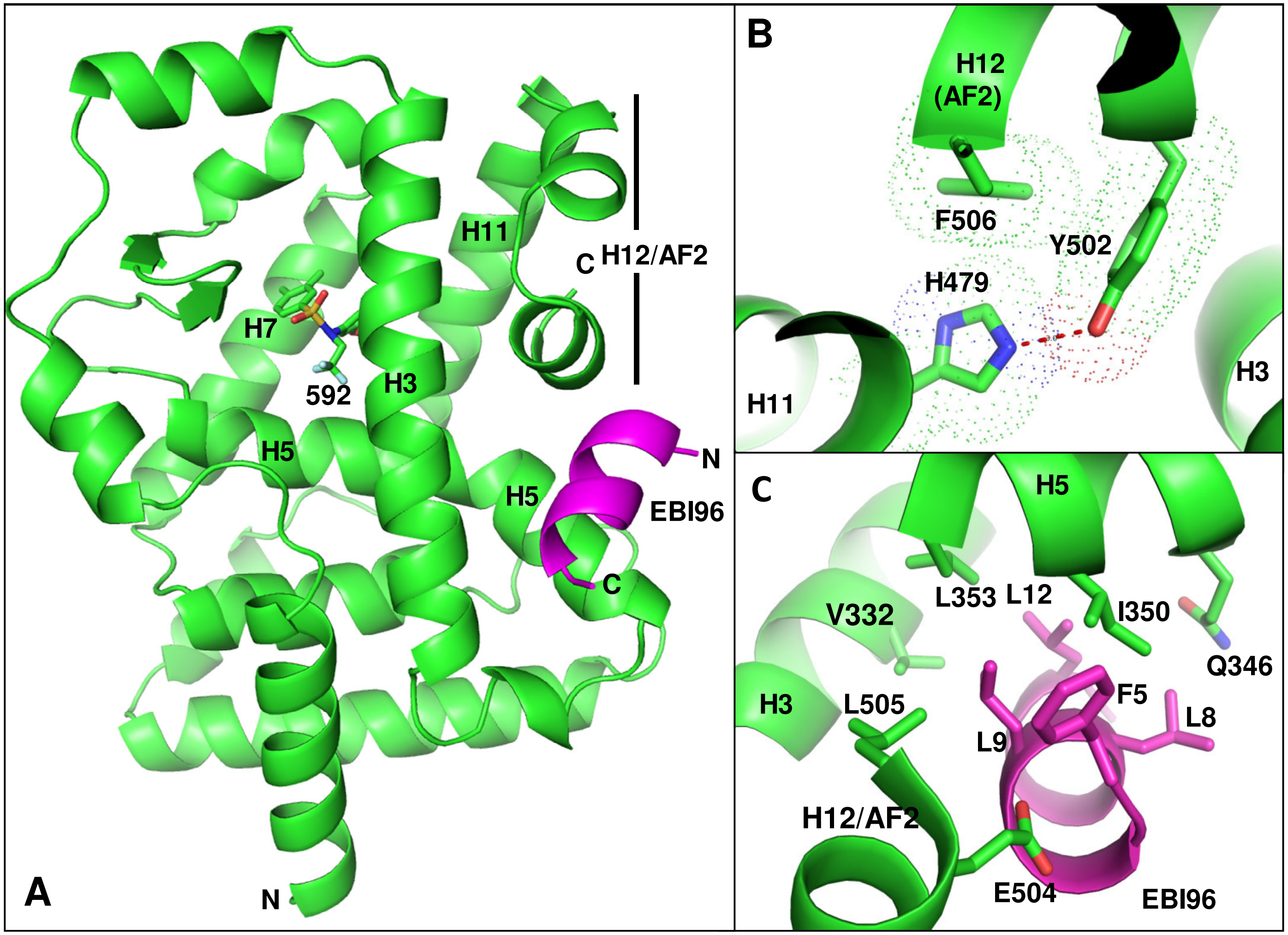

A
H12/AF2
C
H11
H7
592
H3
H5
H5
N
EBI96
C
N
B
H12
(AF2)
F506
Y502
H479
H11
H3
C
H5
L353
L12
I350
V332
Q346
H3
L505
F5
L8
L9
H12/AF2
E504
EBI96

A

H9 H1

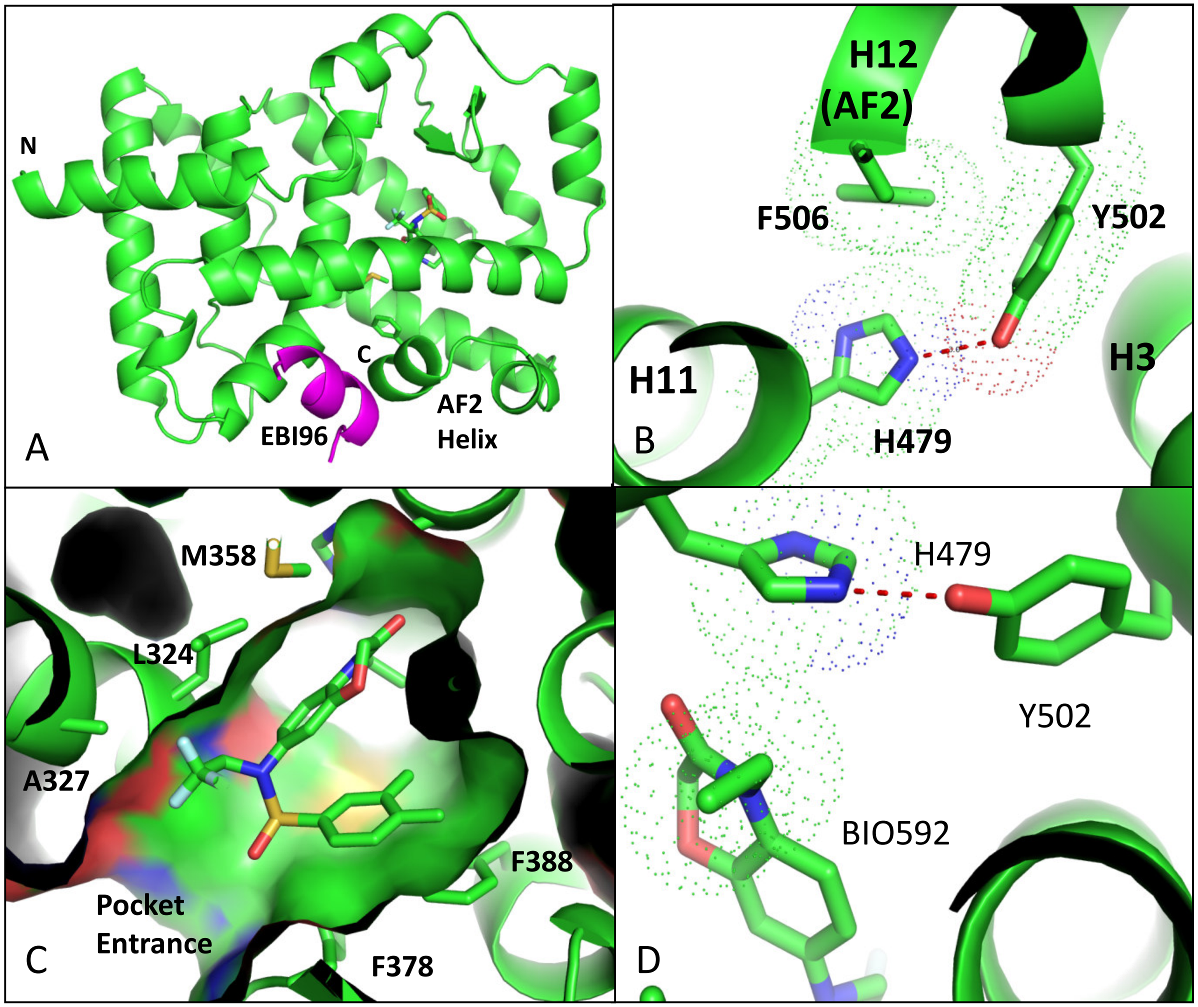
N
C
EBI96
AF2
Helix
A
H12
(AF2)
F506
Y502
H11
H3
H479
B
M358
L324
A327
F388
Pocket
Entrance
F378
C
H479
Y502
BIO592
D

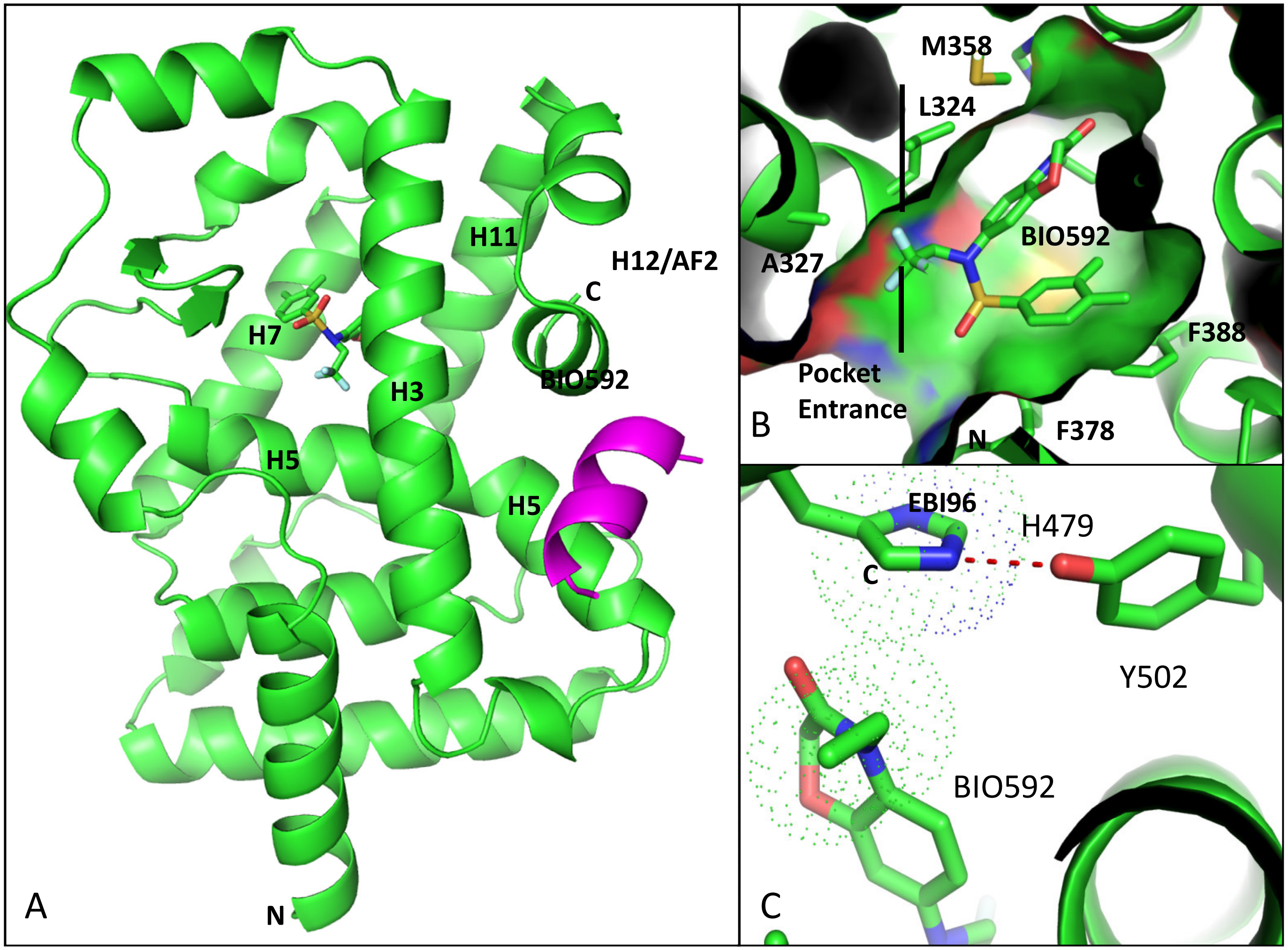

H11
H12/AF2
C
H7
BIO592
H3
H5
H5
N
A
M358
L324
BIO592
A327
F388
Pocket
Entrance
B
N
F378
EBI96
H479
C
Y502
BIO592
C

RORγ518	Actinase E	C-terminal ending (Species %)
APO	-	518 (100)
APO	+	504 (50), 503 (40), 502 (10)
BIO592 + EBI96	+	518 (55), 514 (45)
BIO399	+	493 (44), 494 (33), 492 (10), 495 (6), 503 (3), 500 (2), 504 (2)

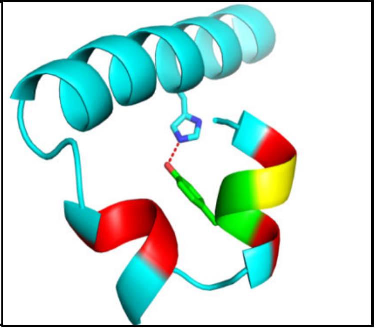

RORγ518	Actinase E	C-terminal Cleavage position (Species %)
**APO**	-	518 (100)
**APO**	+	50 4 (10), 505 (40), 506 (50)
**BIO592 + EBI96**	+	518 (55), 515 (45)
**BIO399**	+	493 (10), 494 (35), 495 (20), 496 (10), 501 (3), 504 (9), 505 (6), 518 (7)

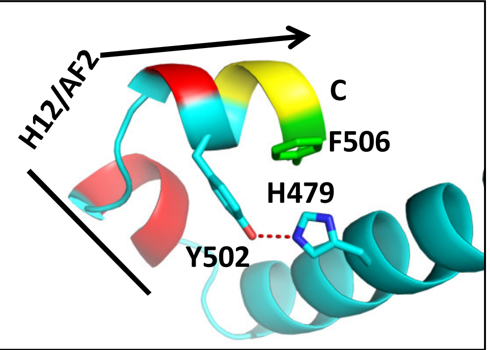

Table 2: Positions of Actinase E proteolysis sites for APO, Ternary BIO592 EBI96 complex and BIO399 determined by mass spectrometry.
Figure3: Positions on the AF2 helix where Actinase E cleaves the APO RORγ (Green), BIO399 complex (Red),sites seen in both (Yellow).

RORγ518	Actinase E	C-terminal Cleavage position (Species %)
**APO**	-	518 (100)
**APO**	+	50 4 (10), 505 (40), 506 (50)
**BIO592 + EBI96**	+	518 (55), 515 (45)
**BIO399**	+	493 (10), 494 (35), 495 (20), 496 (10), 501 (3), 504 (9), 505 (6), 518 (7)

Additional file 5. Positions of Actinase E proteolysis sites for APO, Ternary BIO592 EBI96 complex and BIO399 determined by mass spectrometry.

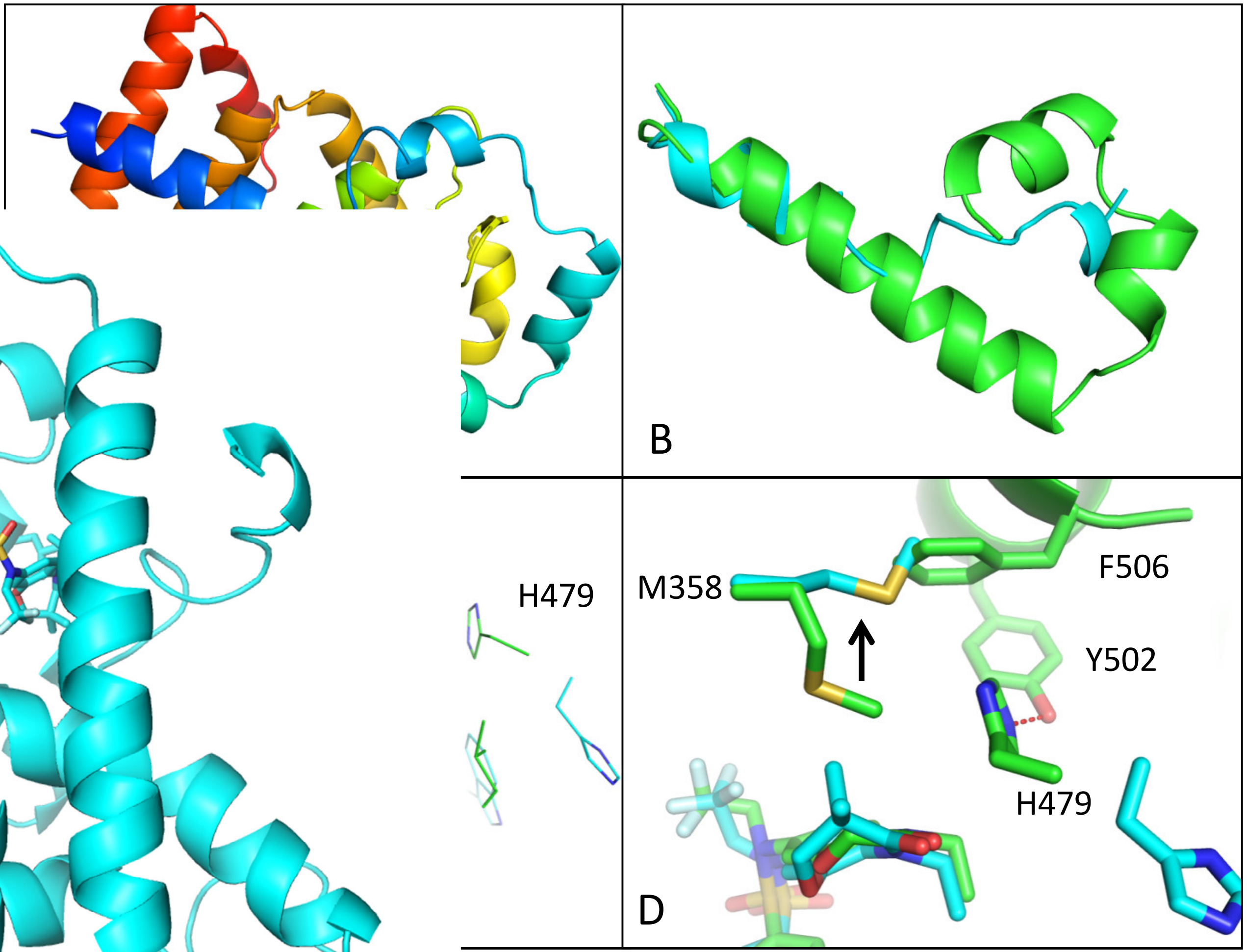

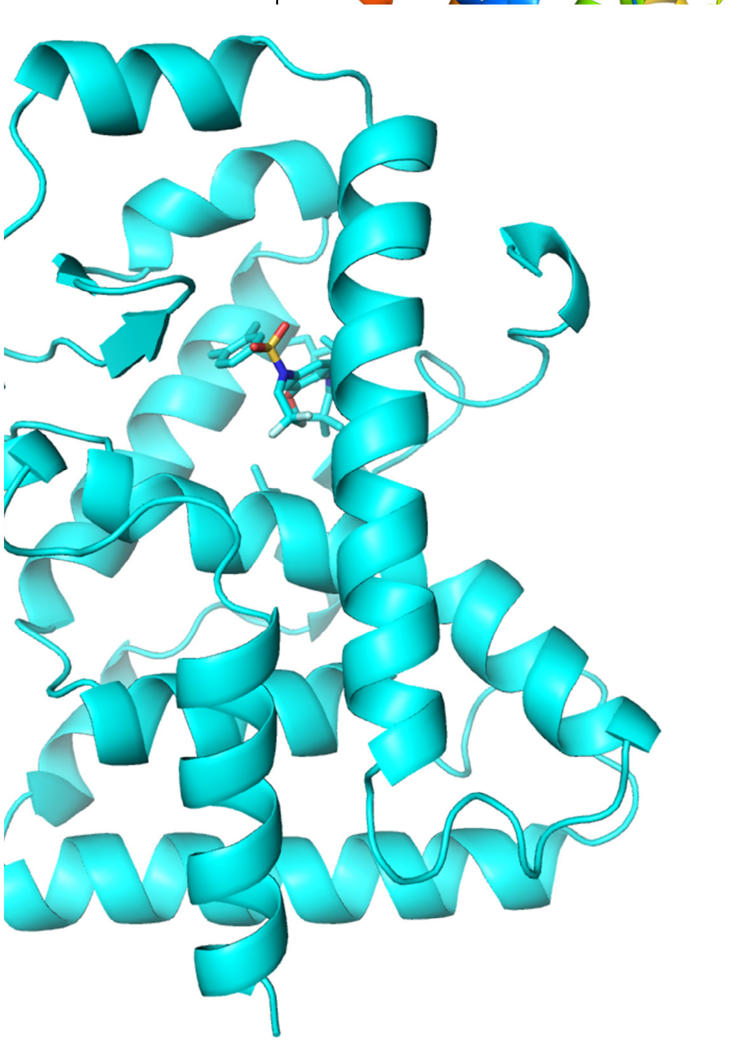

ı93/4 BIO399 complex. B) Comparison of c-termini of
ɖ RORγ493/4 BIO399 (Blue). C) Overlay of BIO592 and
D) Comparison of M358 in BIO592 and BIO399 RORγ

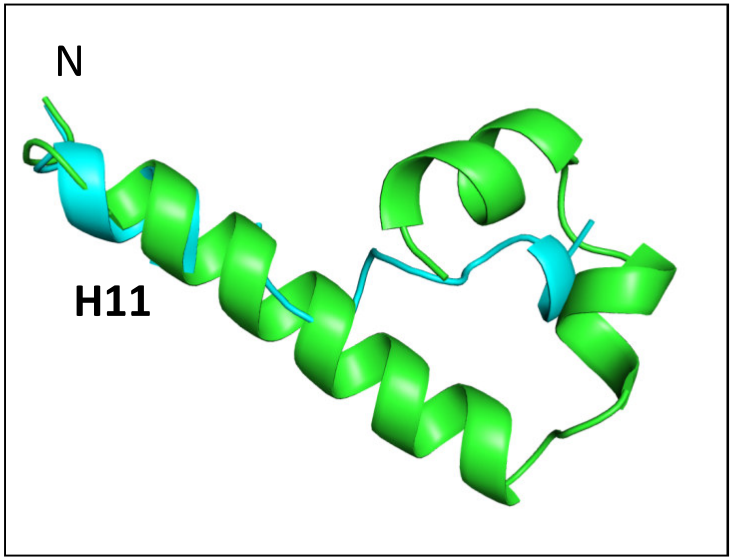
N
H11

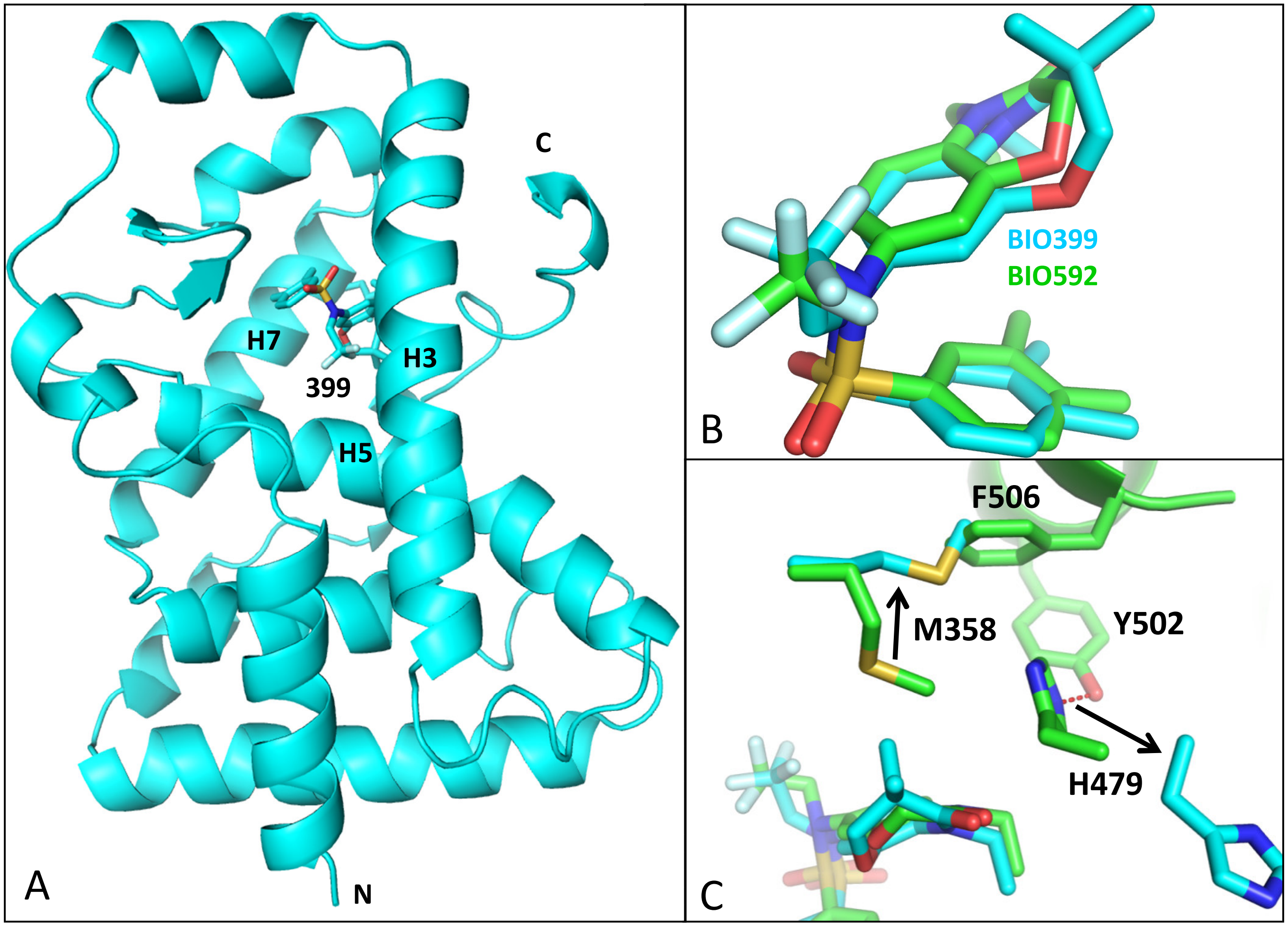
C
H7
399
H3
H5
A
N
BIO399
BIO592
B
F506
M358
Y502
H479
C

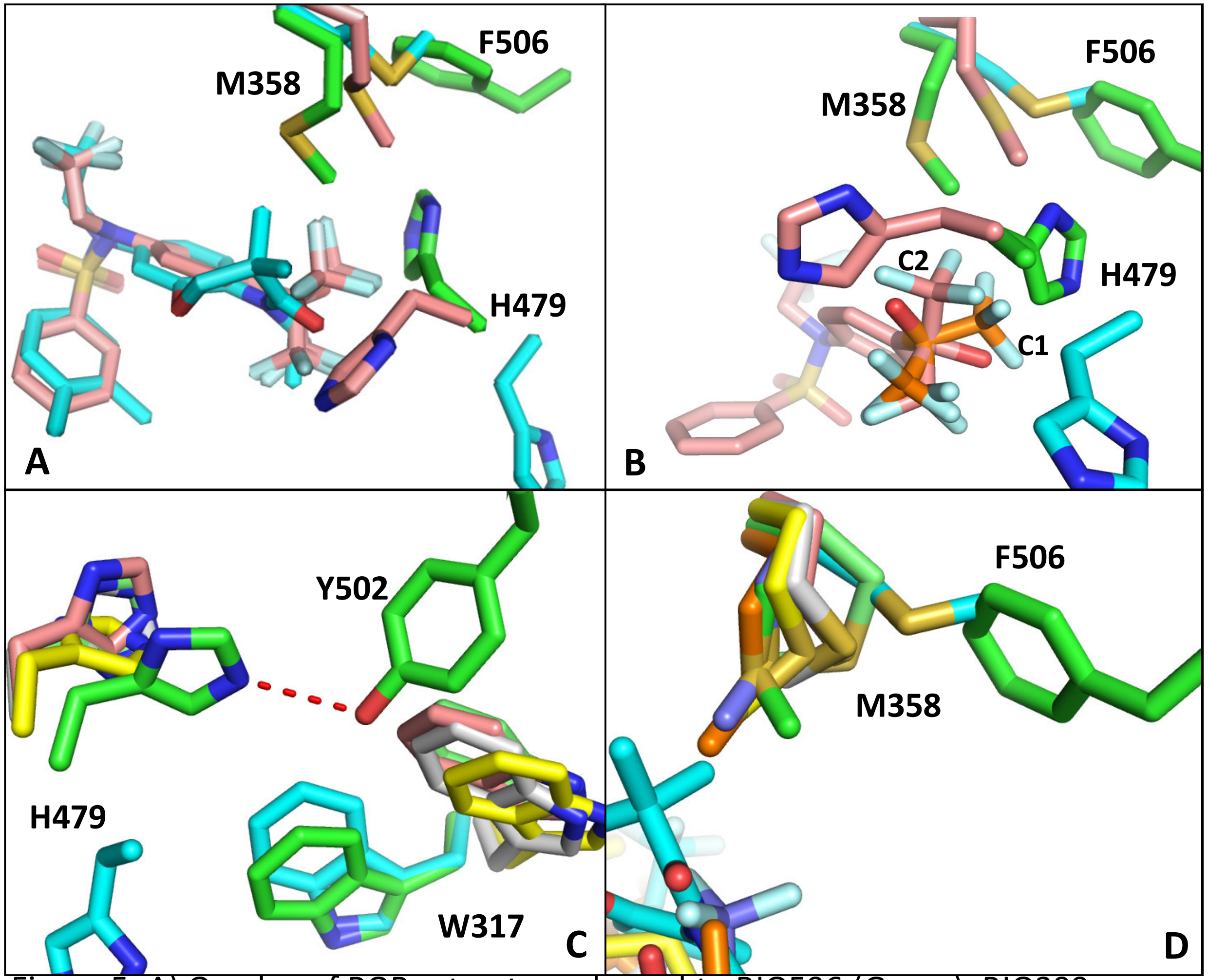

Figure 5: A) Overlay of RORγ structures bound to BIO596 (Green), BIO399 (Cyan) and T0901317 (Pink). B) The two conformations for the the CF_3 on the hexafluropropanol of T0901317 (C1: Orange/ C2: Pink). Overlay of W317/ H479 (C) and M358 (D) in RORγ structures BIO596 (Green), BIO399 (Cyan), Digoxin (Yellow), Compound 2 (grey), Compound 48 (Salmon) and Compound 4j (Lime).

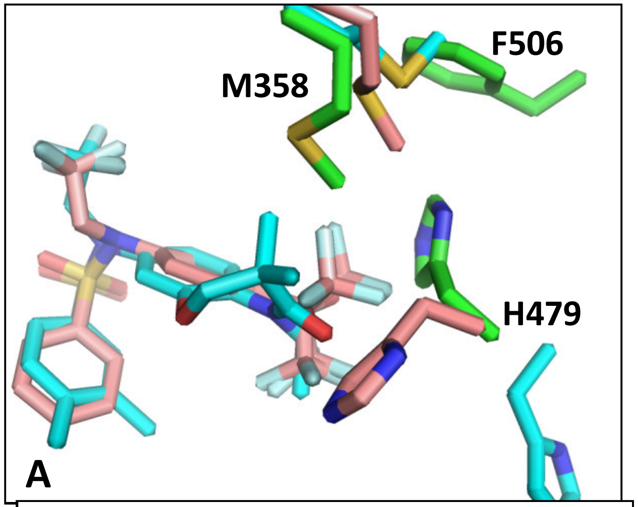

M358

F506

BIO399

B

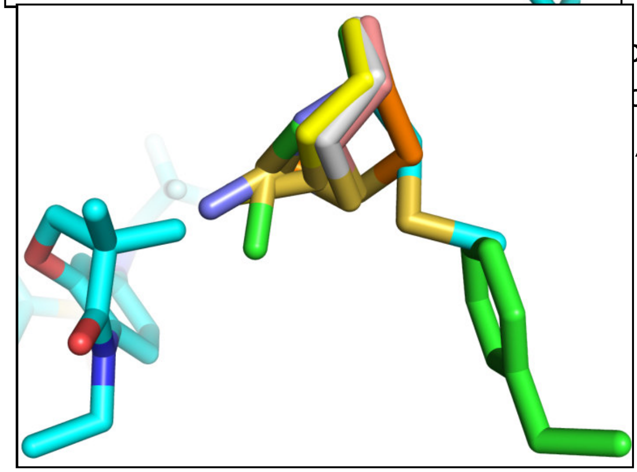

pound to BIO596 (Green), BIO399
of M358 in RORγ structure BIO596
, Compound 2 (grey), Compound 48

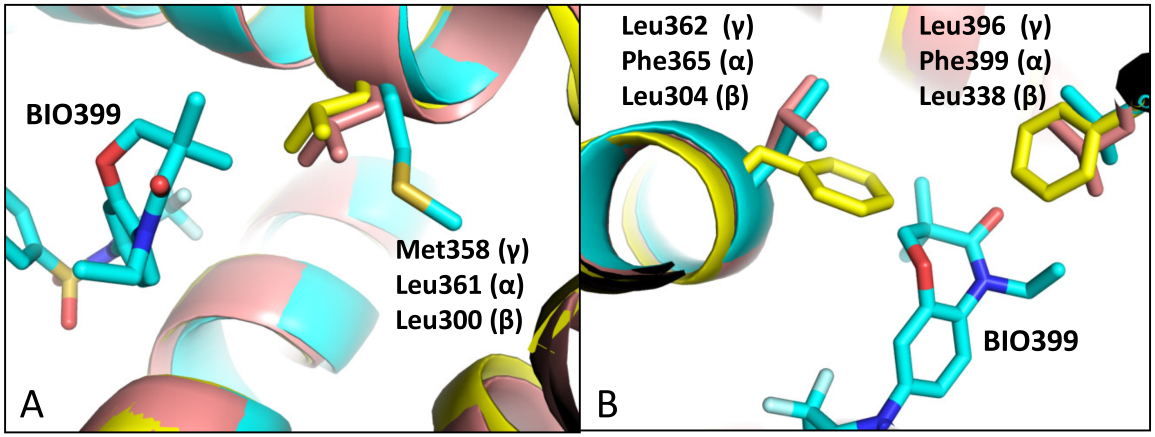

ROR	γ	α	β
EC_{50} (uM)	0.0425	>10	1.2
Selectivity (x)		>235	28.2

Table 3: RORs selectivity profile for BIO399 in GAL4 reporter assay. Figure 6: A) Overlay of RORα,β and γ showing side chain differences at Met358 inverse agonism trigger position(A) and around the benzoxazinone ring system of BIO399 (B).

ROR	EC50 (uM)	Selectivity (X)
γ	0.0425	-
[illegible]	[illegible]	[illegible]

W317 C320 H323 L324 A327M358 V361 M365 V376 F358 F388 L396 I397 I400 F402 H479

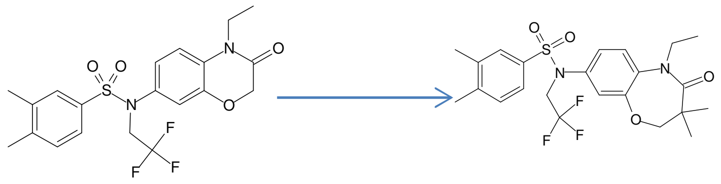
O
O
S
N
N
O
O
F
F
F

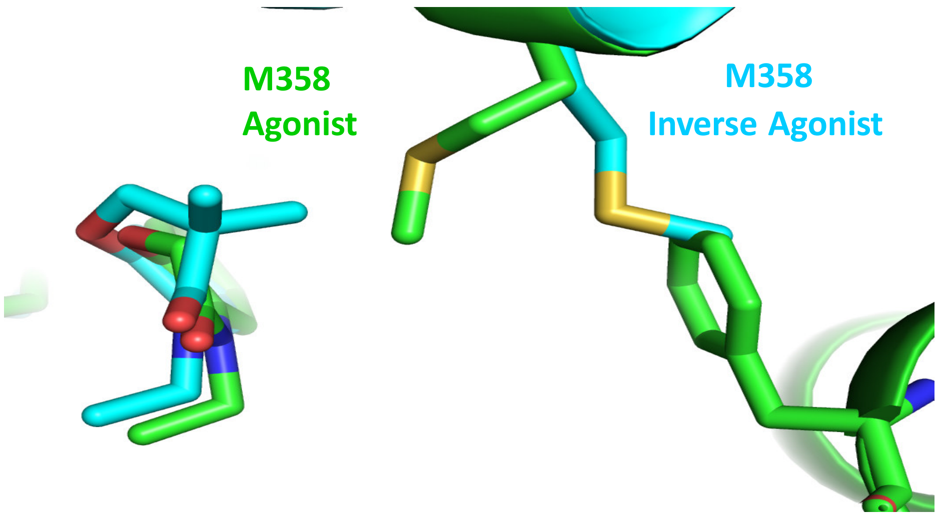
M358
Agonist
M358
Inverse Agonist
