# Supplementary material for: Structural determinant for inducing RORgamma specific inverse agonism triggered by a synthetic benzoxazinone ligand
Source: BMC Struct Biol. 2016 Jun 1;16:7. doi: 10.1186/s12900-016-0059-3 (PMC4888278; doi:10.1186/s12900-016-0059-3)
Supplement: Additional file 5: — Positions of Actinase E proteolysis sites for APO, Ternary BIO592 EBI96 complex and the BIO399 binary complex determined by mass spectrometry. (PDF 30 kb) [file 12900_2016_59_MOESM5_ESM.pdf]

RORγ518	Actinase E	C-terminal Cleavage position (Species %)
**APO**	-	518 (100)
**APO**	+	50 4 (10), 505 (40), 506 (50)
**BIO592 + EBI96**	+	518 (55), 515 (45)
**BIO399**	+	493 (10), 494 (35), 495 (20), 496 (10), 501 (3), 504 (9), 505 (6), 518 (7)

Additional file 4. Positions of Actinase E proteolysis sites for APO, Ternary BIO592 EBI96 complex and the BIO399 binary complex determined by mass spectrometry.
