# Supplementary material for: Structural determinant for inducing RORgamma specific inverse agonism triggered by a synthetic benzoxazinone ligand
Source: BMC Struct Biol. 2016 Jun 1;16:7. doi: 10.1186/s12900-016-0059-3 (PMC4888278; doi:10.1186/s12900-016-0059-3)
Supplement: Additional file 6: — Difference density at 2σ for the position of Met358 in the RORγ molecular replacement model PDB: 3L0L) and refined in the RORγ BIO399 structure. (PDF 343 kb) [file 12900_2016_59_MOESM6_ESM.pdf]

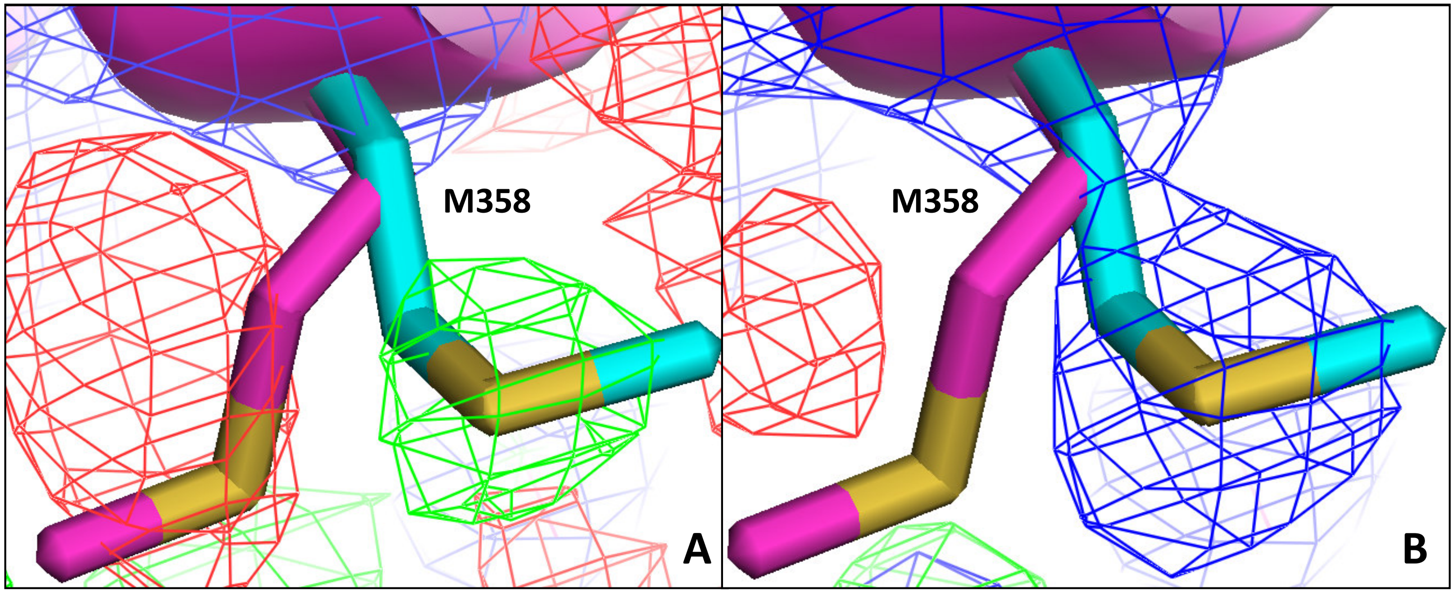

Additional file 6: Position of Met358 in the RORγ BIO399 binary structure. A) Initial difference density at 2σ for Met358 from molecular replacement model 3L0L after first round of refinement (magenta) and alternate rotomer conformation (cyan). B) Electron density for Met358 rotomer after second round of refinement.
