# Supplementary material for: Structural determinant for inducing RORgamma specific inverse agonism triggered by a synthetic benzoxazinone ligand
Source: BMC Struct Biol. 2016 Jun 1;16:7. doi: 10.1186/s12900-016-0059-3 (PMC4888278; doi:10.1186/s12900-016-0059-3)
Supplement: Additional file 8: — Superposition of Trp317, Met358 and His479 side chains in the RORγ BIO592 agonist structure and linear inverse agonist structures. (PDF 525 kb) [file 12900_2016_59_MOESM8_ESM.pdf]

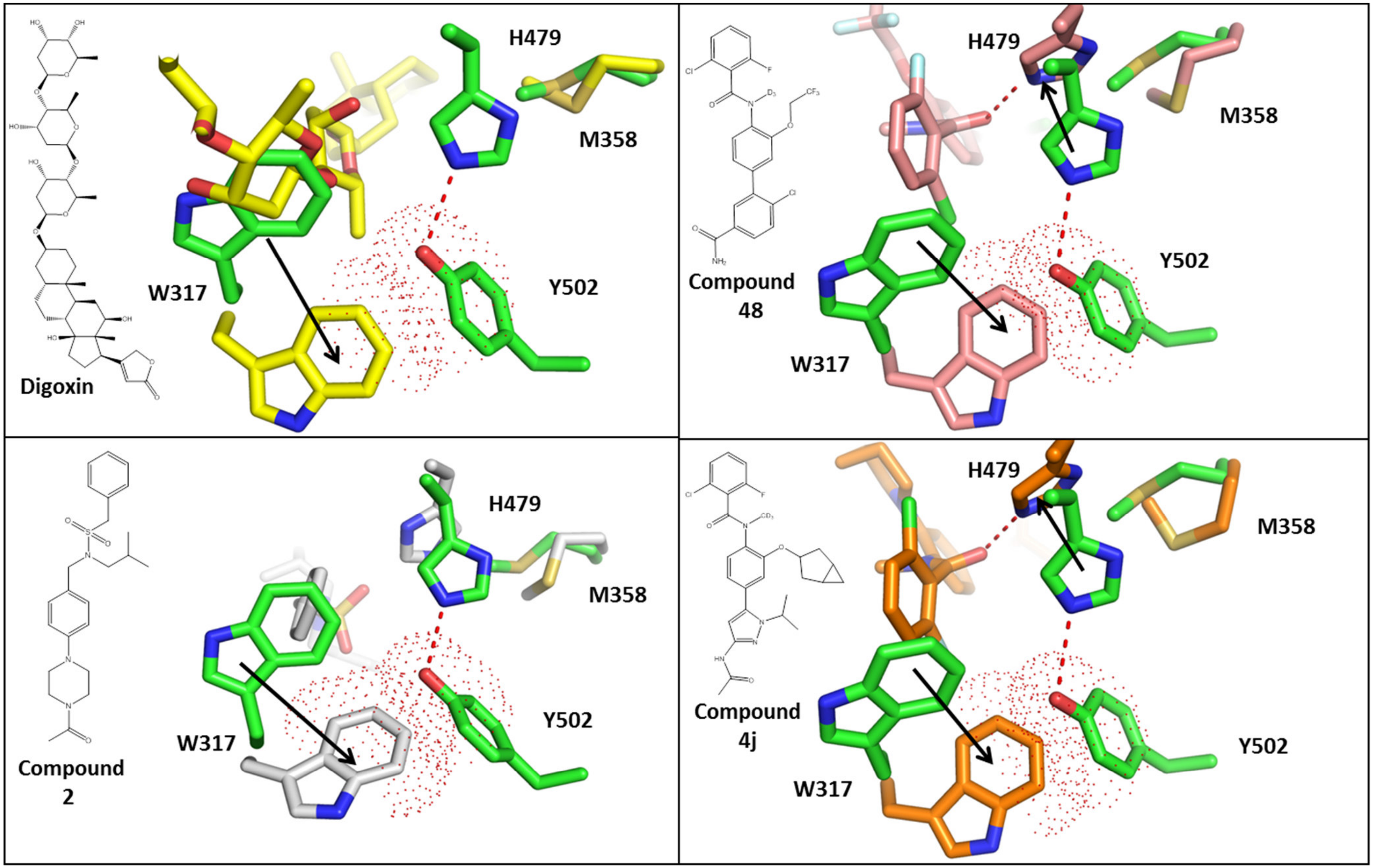

Additional file 8: Overlay of Trp317, Met358 and His479 in RORγ BIO592 agonist structure (green) and linear inverse agonist structures A) Digoxin (yellow (3B0W)), B) Compound 2 (silver (AWQP)), C) Compound 48 (4ZJR (salmon)) and D) Compound 4j (orange (4ZOM)).
